# Lifting eviction moratoria shifted treatment for substance use disorders

**DOI:** 10.1093/haschl/qxag006

**Published:** 2026-01-10

**Authors:** Matthew D Eisenberg, Yimin Ge, Kathryn M Leifheit, Alene Kennedy-Hendricks, Sabriya Linton, Michael Fingerhood, Iraj Qureshi, Leah Robinson, Laken Roberts, Craig Evan Pollack

**Affiliations:** Department of Health Policy and Management, Johns Hopkins Bloomberg School of Public Health, Baltimore, MD 21205, United States; Johns Hopkins Center for Mental Health and Addiction Policy, Baltimore, MD, United States; Department of Mental Health, Johns Hopkins Bloomberg School of Public Health, Baltimore, MD, United States; Department of Health Policy and Management, Johns Hopkins Bloomberg School of Public Health, Baltimore, MD 21205, United States; Johns Hopkins Center for Mental Health and Addiction Policy, Baltimore, MD, United States; Department of Health Policy & Management, University of California, Los Angeles Fielding School of Public Health, Los Angeles, CA, United States; Department of Health Policy and Management, Johns Hopkins Bloomberg School of Public Health, Baltimore, MD 21205, United States; Johns Hopkins Center for Mental Health and Addiction Policy, Baltimore, MD, United States; Department of Mental Health, Johns Hopkins Bloomberg School of Public Health, Baltimore, MD, United States; Johns Hopkins Center for Mental Health and Addiction Policy, Baltimore, MD, United States; Department of Mental Health, Johns Hopkins Bloomberg School of Public Health, Baltimore, MD, United States; Johns Hopkins Center for Mental Health and Addiction Policy, Baltimore, MD, United States; Department of Mental Health, Johns Hopkins Bloomberg School of Public Health, Baltimore, MD, United States; Johns Hopkins School of Medicine, Baltimore, MD, United States; Department of Health Policy and Management, Johns Hopkins Bloomberg School of Public Health, Baltimore, MD 21205, United States; Johns Hopkins Center for Mental Health and Addiction Policy, Baltimore, MD, United States; Department of Health Policy and Management, Johns Hopkins Bloomberg School of Public Health, Baltimore, MD 21205, United States; Johns Hopkins Center for Mental Health and Addiction Policy, Baltimore, MD, United States; Department of Health Policy and Management, Johns Hopkins Bloomberg School of Public Health, Baltimore, MD 21205, United States; Department of Health Policy and Management, Johns Hopkins Bloomberg School of Public Health, Baltimore, MD 21205, United States; Johns Hopkins Center for Mental Health and Addiction Policy, Baltimore, MD, United States; Johns Hopkins School of Medicine, Baltimore, MD, United States; Johns Hopkins School of Nursing, Baltimore, MD, United States

**Keywords:** eviction moratorium, substance use disorder, opioid use disorder, housing policy

## Abstract

**Introduction:**

About 2.7 million U.S. households face an eviction filing each year. The effect of eviction policy on substance use disorder (SUD) treatment is uncertain.

**Methods:**

We used IQVIA all-payer claims, Jan 2020–Dec 2021, in a difference-in-differences design. Phase 1 compared states where moratoria expired March–August 2020 with states that kept protections. Phase 2 examined the August 2021 end of the federal moratorium, contrasting states with ongoing state moratoria vs none. The population was people with a mental health or SUD diagnosis. Outcomes were state-week counts of unique patients with outpatient SUD visits, inpatient SUD stays, medications for opioid or alcohol use disorder, and opioid use disorder (OUD)-specific outpatient and inpatient care.

**Results:**

Ending moratoria was associated with more patients receiving outpatient SUD care: +3.3% (95% CI −0.5% to 7.2%; *P* = 0.09) in Phase 1 and +4.9% (95% CI 1.8% to 8.0%; *P* = 0.002) in Phase 2. We observed no change in inpatient SUD care. In Phase 2, medication treatment increased by 2.5% (95% CI 0.3% to 4.7%; *P* = 0.03). OUD-specific results were similar.

**Conclusion:**

The return of eviction risk coincided with greater use of clinic-based SUD services but not hospital care. Housing policy may shift where and how people seek SUD treatment.

## Introduction

Stable housing supports treatment for substance use disorders (SUD) because it removes the stress of a potential move, protects social networks, and makes it easier to keep appointments among other mechanisms.^1-3^ At the other extreme, homelessness is associated with increased environmental cues for substance use, uncomfortable and unsafe living conditions, and decreased ability to access treatment.^4^ Individuals experiencing homelessness may also report taking stimulants in particular to stay vigilant against violence and other threats to safety.^4,5^ Evictions (forced displacement from housing via legal means) are associated with increased overdose risk in quantitative^6,7^ and qualitative research.^8^ However, the impact of policies designed to reduce eviction risk on engagement with treatment for SUDs remains largely unknown.

Pandemic-era eviction moratoria, or bans, offer a natural experiment to measure these effects. COVID-19 triggered sharp job losses,^9,10^ and many renters fell behind on housing payments.^11,12^ In response, most states issued emergency orders in March and April 2020 that stopped new eviction filings or halted enforcement of existing judgments for any period of a few weeks to several months.^13^ On September 4, 2020, the Centers for Disease Control and Prevention (CDC) set a national moratorium that filled the gaps left by expiring state actions,^14^ protections which remained in place until August 26, 2021.^13^ After the Supreme Court ruling overturning the CDC moratorium, nine states kept their own moratoriums in force, while the rest allowed evictions to resume immediately.

Despite documented links between evictions, substance use, and healthcare utilization, it remains unknown how these major policy changes impacted SUD treatment. However, the role of SUD treatment in this association remains unclear. Prior research indicates that eviction moratoria reduced immediate fears of needing to move and was associated with improved mental health.^15-19^ Thus, resumption of evictions might increase stress, problematic substance use, and demand for treatment.^20^ On the other hand, because evictions are associated with interruptions in preventive medical care,^21^ expiration of moratoria might also be associated with a decrease in SUD treatment engagement. By leveraging expiring moratoria as a natural experiment, we can understand the *net* impact of increasing population-level eviction burden on SUD service utilization, taking into consideration these two competing mechanisms. No national study has connected the timing of moratorium expirations to objective measures of outpatient visits, inpatient stays, and medication fills. Our work fills this gap by using comprehensive all-payer claims and a difference-in-differences design across two distinct policy windows,^22,23^ to estimate the impact of eviction moratoria expiration on the treatment of SUD.

## Methods

### Data and sample

We extracted data from two national, all-payer IQVIA files.^24,25^ The Longitudinal Prescription (LRx) database logs about 3.7 billion retail, mail-order, and long-term-care pharmacy transactions each year, covering an estimated 90% of U.S. retail fills, 75% of mail-order fills, and 75% of long-term-care fills for roughly 250 million patients. Each record includes the drug dispensed, dose, days supplied, prescriber, payer, and patient ZIP code. The Medical Claims (Dx) database contains pre-adjudicated professional claims submitted on CMS-1500 or 837P forms (i.e. standard health care claims forms) through clearinghouses and practice-management software, capturing diagnoses, procedures, tests, and drugs ordered during encounters at office-based practices, opioid treatment programs, ambulatory centers, hospitals, and skilled-nursing facilities. Pre-adjudicated claims are bills submitted by clinicians to payers before the payer has finalized the payment amount. While they contain diagnosis and procedure codes, they precede final payment or denial. The Dx database's contains data on approximately 191 million patients.

The dataset built from these two IQVIA data sources included every person with ≥1 claim listing a mental health or substance use diagnosis between 2015 and 2022; all medical and pharmacy claims linked to those patients were retained (see Appendix Table S1). We further limited the sample by keeping claims only from clinicians who appeared in the data at least once in every calendar year from 2019 through 2022, to avoid bias from providers that enter and exit IQVIA's data collection frame. We also dropped provider-year observations whose claim counts exceeded the 99th percentile, as these are likely data errors and not individual providers. State was taken from the patient address recorded on each claim and updated when a person moved. Data were aggregated up to the state-week level measure outcomes for the period between March 1st 2020 and December 31st 2021.

### Outcome measures

For every U.S. state and each calendar week in 2020–2021, we counted the number of unique patients with (1) any outpatient SUD visit and (2) any inpatient SUD stay. Outpatient vs inpatient encounters were defined using the place-of-service code (Appendix Table S2). Outpatient visits include both in-person and telehealth encounters in office/clinic ambulatory settings, but we could not consistently distinguish telehealth from in-person visits over time because telehealth billing codes and modifiers were not used consistently across payers and states. A claim was classified as SUD if it included an ICD-10 diagnosis code F10-F19 (excluding F17.2x) which encompassed use disorders related to opioids, cannabis, alcohol, cocaine, stimulants, sedative/hypnotic, cocaine, hallucinogens, and inhalants. SUD codes were required to appear in the primary diagnosis field, or in a secondary field if the primary diagnosis was for a mental health condition (F20-F69, F84, F90-F99). The next outcome was the number of individuals (3) receiving an FDA-approved medication for opioid or alcohol use disorder (the only SUD diagnoses with FDA-approved medications) including both procedure codes for medicine administration and National Drug Codes for medicine dispensing (Appendix Tables S3 and S4). Medicines included formulations of buprenorphine, methadone, naltrexone, acamprosate, and disulfiram. Given the importance of opioid use disorder (OUD), we also measured number of individuals with: (4) any OUD outpatient visits and (5) any OUD inpatient visits using ICD-10 code F11.

### Explanatory variables

**Figure 1.**
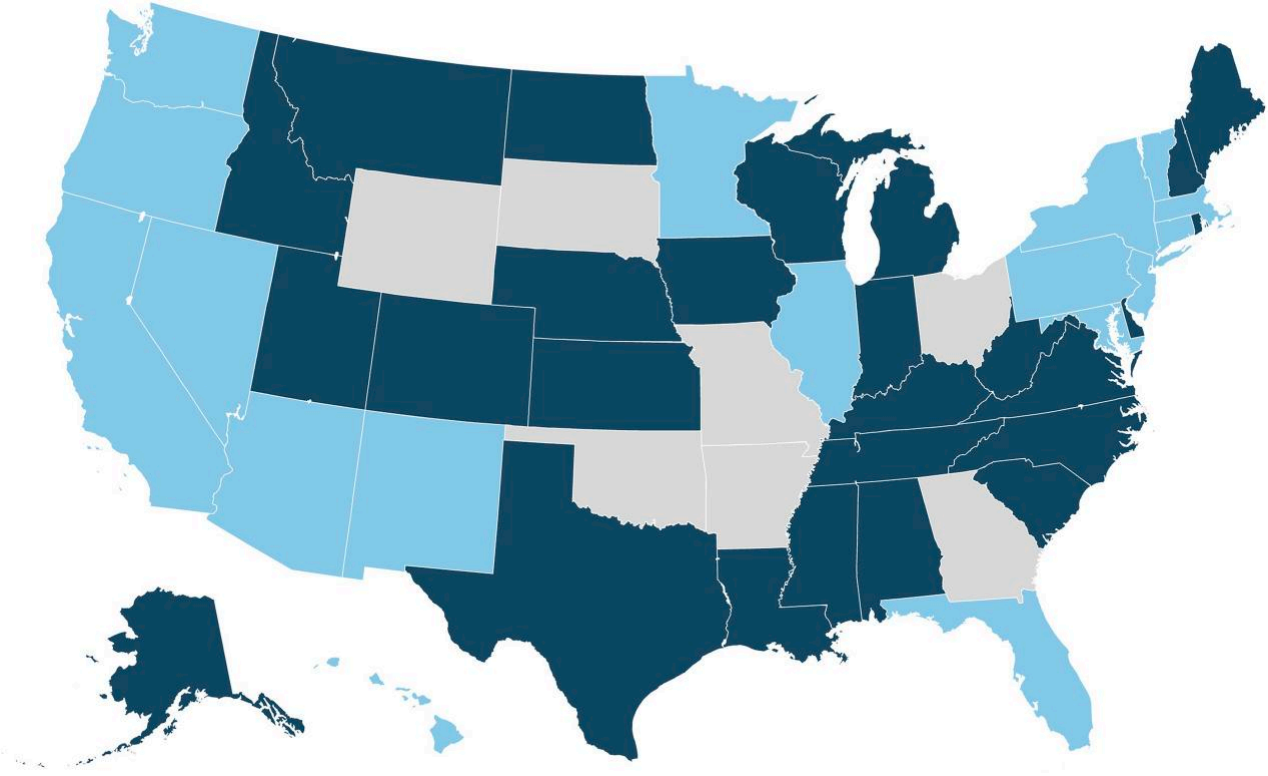
Phase 1: March–August 2020. *Notes:* Dark blue = Expired. Light blue = Maintained. Gray = N/A. In the Phase 1 analysis, the treatment is the expiration of state-level eviction moratorium expiration of states in the treatment group. States in the control group include those that had state-level moratoriums but their moratorium did not expire during our observation period. Gray states never had a state based moratorium during Phase 1 and are excluded from our analysis.

**Figure 2.**
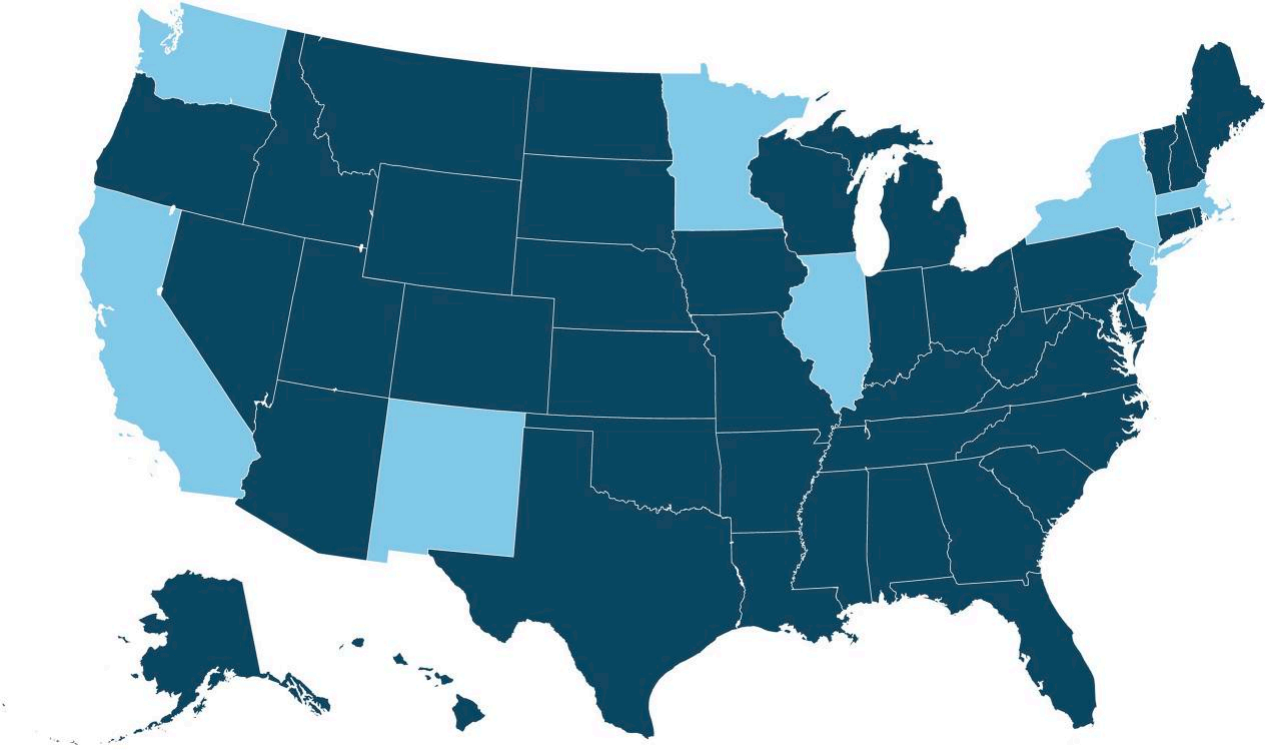
Phase 2: March–December 2021. Notes: Dark blue = Expired. Light blue = Maintained. In the Phase 2 analysis, the treatment is the expiration of federal-level eviction moratorium expiration. States in the control group include those that continued to have state-level moratoriums after the expiration of the federal moratorium.

Using the state-week panel dataset spanning March 2020–December 2021, we separately evaluated two policy shocks: (1) the staggered expiration of state eviction moratoria in 2020, before the nationwide CDC moratorium began and (2) the expiration of the federal moratorium in 2021.^26^ Each shock was analyzed separately because the composition of treated and comparison states differed and policy environments changed over time (see Figures 1 and 2).In Phase 1 (March 11th–September 1st, 2020), states entered the sample when their eviction moratoriums went into effect (44 states total). Treated states were those with a state eviction moratorium that expired during this period; comparison states were those retaining their moratorium for the entire period. We excluded state-week observations extending beyond 12 weeks before ban expiration and 16 weeks after ban expiration given that few states had data beyond this period. In Phase 2 (Jun 4th–Dec 23rd, 2021), the exposure was the Supreme Court ruling on August 26th, 2021 that ended the CDC-issued federal moratorium, triggering evictions to resume in states without an active state-level moratorium (i.e., treated states; *n* = 42). Nine jurisdictions, California, the District of Columbia, Illinois, Minnesota, New Jersey, New Mexico, New York, Oregon, and Washington, kept state-level bans in place and served as the comparison group. Our Phase 2 design, focused on the end of the federal moratorium and comparing states that did vs did not maintain state protections afterward, is less linked to the early reopening period and should be less affected by concurrent changes in COVID public-health policies.

### Covariates

While our state fixed effects account for all time invariant state level characteristics, we account for other time-varying pandemic era policies that may impact SUD treatment seeking. We added weekly state indicators for Supplemental Nutrition Assistance Program (SNAP) emergency allotments^27^ and enhanced unemployment insurance payments given known associations between SUD and unemployment. To account for illicit drug-market shifts, we controlled for state indicators of fentanyl seizure volume, measured over 6-month periods.^28,29^ We controlled for Medicaid expansion status which is associated with care seeking. In Phase 2 models, we also adjusted for cumulative, per capita disbursement of emergency rental assistance by state-month. Emergency rental assistance was a 2021 policy which reduced eviction risk^30^ by absolving rental debts accrued during the pandemic and thus may have buffered potential adverse effects of expiring eviction moratoriums.

### Statistical analysis

For Phase 1, we applied the Callaway-Sant’Anna difference-in-differences estimator,^31^ which handles staggered policy (de-)adoption by comparing each state before and after the moratorium lapsed with states whose bans were still in force during the same week. We used augmented inverse probability weighting (AIPW) to minimize threats from model misspecification and use the “never-treated” states as the comparison group. Event-study coefficients were estimated using a single common reference period (the week immediately before expiration), using the **long2** option in **csdid** (STATA/MP18), so each lead/lag is interpreted relative to the same baseline week.

Phase 2 used a two-way fixed-effects Poisson model with a single indicator for weeks after the federal moratorium ended in treated states. Our primary estimand was the short-run change in SUD treatment utilization in the 16 weeks after moratorium expiration (when eviction risk resumed) among individuals with a pre-existing burden of SUD and mental health conditions, rather than the full process from new onset substance use to treatment initiation.

All models included log-transformed outcomes to correct the right-skewed distribution. We also estimated event study specifications to confirm that treated and comparison states followed similar trends before expiration. All models contained state and year-week fixed effects, were weighted by state population in order to better estimate effects for the country overall (rather than unweighted models which give equal weight to very small and very large states) and employed heteroskedasticity robust standard errors clustered at the state level. Analyses were run in Stata/MP 18, and two-sided *P* values < 0.05 were considered statistically significant.

We ran several key sensitivity analyses. First, in Phase 1 analyses, we omitted any state-week in which more than 10% of residents were still shielded by a city or county ban after the state-level moratorium had ended.^32^ This step tests whether large local orders could blur our measurement of treatment timing. Second, we re-estimated our models with a COVID policy stringency index^33^ to better control for things like school closures, workplace closures etc. Third, to test the sensitivity of our models to co-occurring policies, we re-estimate our models removing the concurrent policy control variables. Fourth, to test if our results are being driven by changes in insurance coverage or changes in sample composition, we estimate models regressing our exposures on the share of insurance types (e.g., share Medicaid, share commercial) and demographics (e.g., share female, average age).

We also estimated three specification checks to ensure our modeling choices were robust. First, we repeated every model without population weights to see whether estimates were driven by the biggest states rather than by a consistent pattern across the country. Second, because Phase 2 patient counts showed extra dispersion, we re-estimated this model with a negative binomial link, which allows the variance to exceed the mean. Third, given small cells in some sub-experiments in the Callaway Sant’Anna model, we re-estimated our model with other staggered difference-in-difference methodologies including Wooldridge (2023)^34^ and Gardner (2024).^35^

## Results

Phase 1 included 26 treated states and 18 comparison states whose state moratoria did not expire during the window. Phase 2 included 42 treated states that lost protections when the federal order ended and 9 comparison states that kept state-level moratoria. Full summary stats are available in Appendix Tables S5 and S6.

In Phase 1, treated states averaged 1028 patients receiving outpatient SUD care per week while bans were in force and 998 patients after expiration. Comparison states averaged 2419 patients receiving SUD care per week over the entire time period. Expiration was associated with a 3.3% relative higher number of patients receiving weekly outpatient SUD care (95% CI, −0.5% to 7.2%; *P* = 0.09; Table 1). This means that even though the number of patients was decreasing in both the treatment and control group, they were decreasing more slowly in the treatment group, leading to a *relative* increase. In Phase 2, treated states averaged 1504 patients receiving outpatient SUD care per week before the federal order ended and 1499 after, while comparison states averaged 3629 and 3565. The end of the federal order was associated with a 4.9% relative increase (95% CI, 1.8% to 8.0%; *P* = 0.002; Table 2) (event studies in Appendix Figures S1 and S2).

**Table 1.** Percent change in outcomes attributable to eviction moratorium expiration during Phase 1 (Mar 11th–Sep 1st).

	*N*	Pre-period (mean)	Post-period (mean)	Percent change (95% CI)	*P*-value	Percent change (95% CI) (Graphical)
**SUD outpatient**						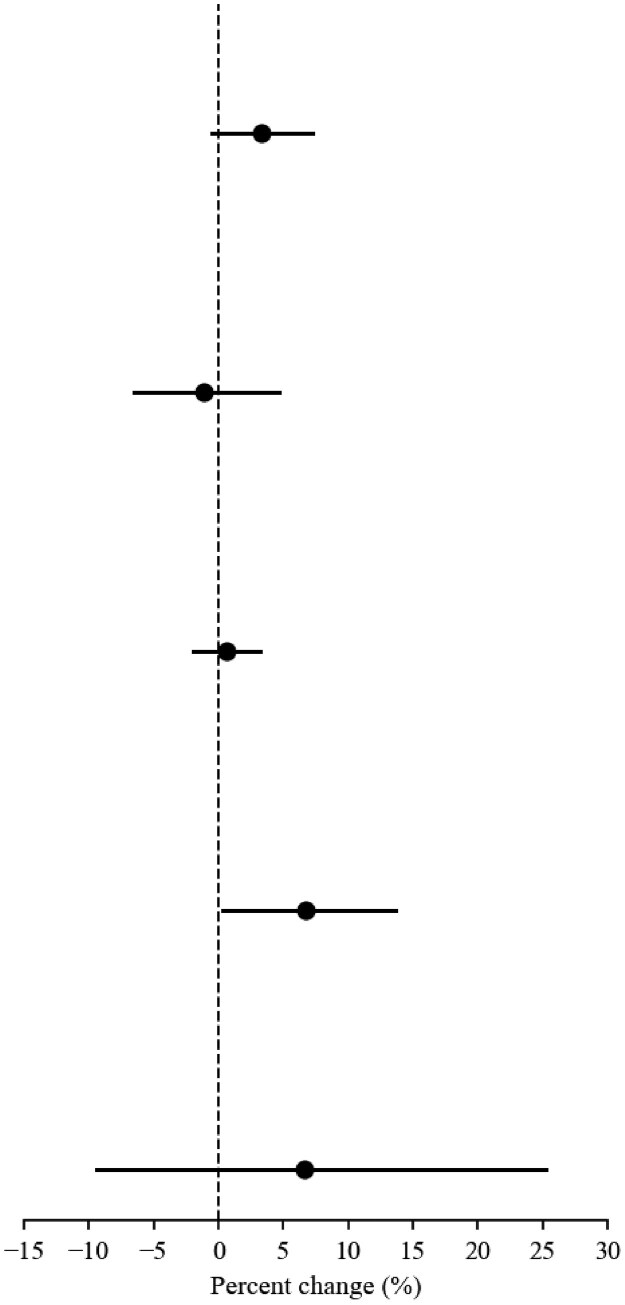
Treated States	26	1028	998	3.28% (−0.50%, 7.20%)	0.090	
Untreated States	18	2419				
**SUD inpatient**						
Treated States	26	170	167	−1.10% (−6.49%, 4.61%)	0.699	
Untreated States	18	390				
**SUD medication**						
Treated States	26	2609	2286	0.58% (−1.97%, 3.19%)	0.659	
Untreated States	18	5634				
**OUD outpatient**						
Treated States	26	450	411	6.72% (0.29%, 13.59%)	0.040	
Untreated States	18	878				
**OUD inpatient**						
Treated States	26	24	22	6.56% (−9.37%, 25.26%)	0.442	
Untreated States	18	59				

In the Phase 1 analysis, Callaway & Sant’ Anna's estimation method is used for analysis. The log version of the three outcomes is taken for estimation. Outcomes are state-week counts of unique patients with at least one qualifying claim in that calendar week (2020–2021). Outpatient vs inpatient encounters were defined using place-of-service codes (Appendix Table S2); outpatient visits include in-person and telehealth office/clinic ambulatory encounters. A claim was classified as SUD if it included an ICD-10 diagnosis code F10–F19 (excluding F17.2x), appearing in the primary diagnosis field, or in a secondary field when the primary diagnosis was a mental health condition (F20–F69, F84, F90–F99). Medication outcomes capture receipt of FDA-approved medications for opioid or alcohol use disorder; OUD outcomes were defined analogously but restricted to ICD-10 code F11. The treatment is the expiration of the state-level eviction moratorium policy. The data sample includes 44 states having state-level eviction moratoriums. The study period is from week 11 to week 35 in 2020, starting from the week when the state's eviction moratorium started. The treatment is the expiration of state-level eviction moratorium expiration of states in the treatment group. States in the control group include those that had state-level moratoriums but their moratorium did not expire during our observation period. The number of observations is 979 for each. State and year-month fixed effects are included.

**Table 2.** Percent change in outcomes attributable to eviction moratorium expiration during Phase 2 (Jun 4th–Dec 23rd, 2021).

	*N*	Pre-period (mean)	Post-period (mean)	Percent change (95% CI)	*P*-value	Percent change (95% CI) (Graphical)
**SUD outpatient**						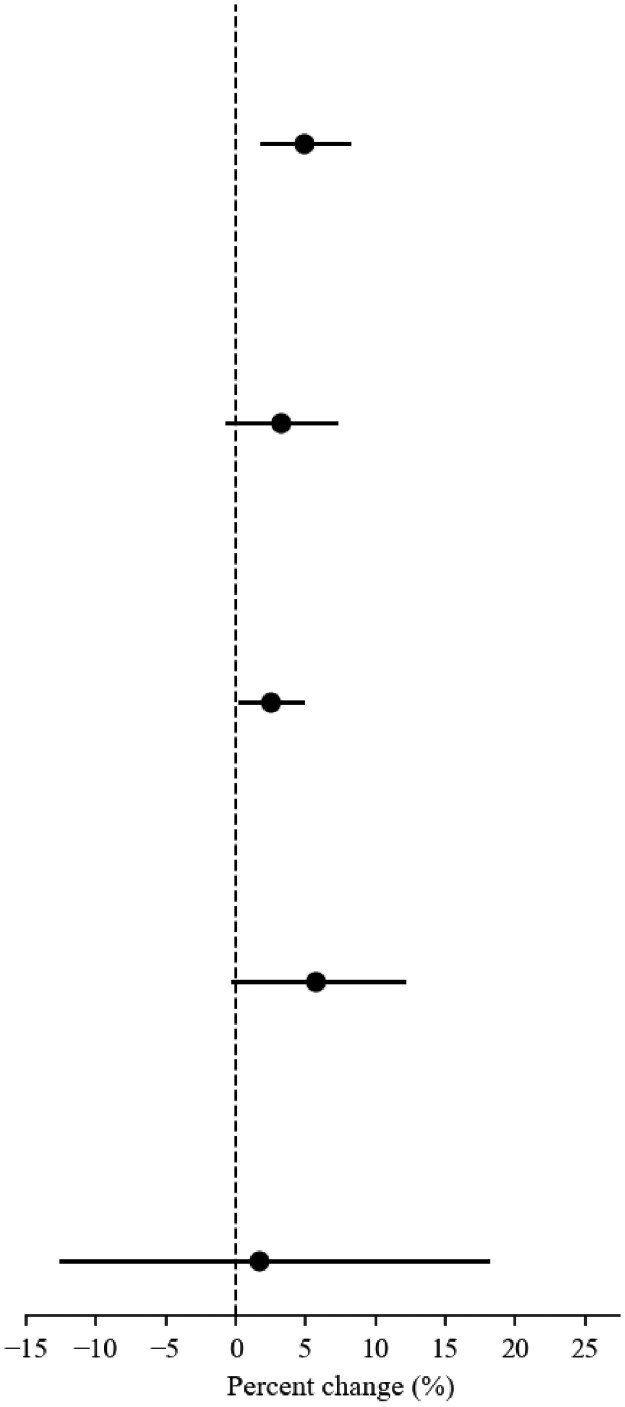
Treated States	42	1504	1499	4.87% (1.81%, 8.02%)	0.002	
Untreated States	9	3629	3565			
**SUD inpatient**						
Treated States	42	211	192	3.15% (−0.65%, 7.09%)	0.106	
Untreated States	9	581	541			
**SUD medication**						
Treated States	42	3386	3506	2.48% (0.27%, 4.74%)	0.028	
Untreated States	9	7502	7432			
**OUD outpatient**						
Treated States	42	623	631	5.72% (−0.21%, 12.01%)	0.059	
Untreated States	9	1305	1306			
**OUD inpatient**						
Treated States	42	31	28	1.61% (−12.54%, 18.01%)	0.834	
Untreated States	9	93	87			

In the Phase 2 analysis, a standard difference-in-difference method is used for analysis. The Poisson model is used for estimation. Outcomes are state-week counts of unique patients with at least one qualifying claim in that calendar week (2020–2021). Outpatient vs inpatient encounters were defined using place-of-service codes (Appendix Table S2); outpatient visits include in-person and telehealth office/clinic ambulatory encounters. A claim was classified as SUD if it included an ICD-10 diagnosis code F10–F19 (excluding F17.2x), appearing in the primary diagnosis field, or in a secondary field when the primary diagnosis was a mental health condition (F20–F69, F84, F90–F99). Medication outcomes capture receipt of FDA-approved medications for opioid or alcohol use disorder; OUD outcomes were defined analogously but restricted to ICD-10 code F11. The treatment is the expiration of the federal eviction moratorium policy on August 26, 2021. The data sample includes 42 states in the treatment group and 9 states in the control group, with IL and WA censored periods after state-level eviction moratorium expiration. The study period is from week 23 to week 51 in 2021. States in the control group include those that continued to have state-level moratoriums after the expiration of the federal moratorium. The number of observations is 1459 for each. State and year-month fixed effects are included.

For inpatient SUD, treated states in Phase 1 averaged 170 patients with encounters per week during the ban and 167 after, vs 390 in comparison states. The estimated change was −1.1% (95% CI, −6.5% to 4.6%; *P* = 0.70; Table 1). In Phase 2, treated states averaged 211 patients with encounters per week before federal expiration and 192 after; comparison states averaged 581 and 541. The difference-in-differences estimate was 3.2% (95% CI, −0.7% to 7.1%; *P* = 0.11; Table 2).

For SUD medications, treated states in Phase 1 averaged 2609 patients with medication events per week during the ban and 2286 after, compared with 5634 in comparison states. The estimated change was 0.6% (95% CI, −2.0% to 3.2%; *P* = 0.66; Table 1). In Phase 2, treated states averaged 3386 patients with medication events per week before federal expiration and 3506 after; comparison states averaged 7502 and 7432. The federal expiration was associated with a 2.5% relative increase (95% CI, 0.3% to 4.7%; *P* = 0.03; Table 2).

Similar patterns of results were observed when limiting the outcomes to patients receiving OUD care. For outpatient OUD, treated states in Phase 1 averaged 450 patients per week during the ban and 411 after, while comparison states averaged 878. Expiration was associated with a 6.7% relative increase (95% CI, 0.3% to 13.6%; *P* = 0.04; Table 1). In Phase 2, treated states averaged 623 patients per week before federal expiration and 631 after; comparison states averaged 1305 and 1306. The estimate was 5.7% (95% CI, −0.2% to 12.0%; *P* = 0.06; Table 2) (event studies in Appendix Figures S3 and S4).

For inpatient OUD, treated states in Phase 1 averaged 24 patients with an inpatient stay per week during the ban and 22 after, compared with 59 in comparison states. The estimate was 6.6% (95% CI, −9.4% to 25.3%; *P* = 0.44; Table 1). In Phase 2, treated states averaged 31 patients with a stay per week before federal expiration and 28 after; comparison states averaged 93 and 87. The estimate was 1.6% (95% CI, −12.5% to 18.0%; *P* = 0.83; Table 2).

### Sensitivity analyses

Results were robust when excluding the two states which had weeks with more than 10% of the state population under a local (city or county) moratorium, following expiration of the state moratorium (Phase 2 only as Phase 1 did not have any states that met this criteria, Appendix Table S7) and when we added additional COVID policy stringency controls (Appendix Tables S8 and S9). When we explored robustness to removing control variables, we saw no difference when removing SNAP and unemployment insurance variables. There was a slight tempering of some results when removing ERA controls, but the results were qualitatively similar (Appendix Table S10). We observed no relationship between our difference-in-difference policy variables and the share of different insurance types (Appendix Table S11), or, demographic differences (Appendix Table S12).

### Specification checks

The main results were similar when we (1) re-estimated models without population weights (Appendix Tables S13 and S14), (2) using negative binomial instead of Poisson regression (Phase 2 only as Phase 1 used Callaway Sant’Anna Method, Appendix Table S15), and (3) tested sensitivity to the Gardner^35^ and Wooldridge^34^ staggered difference-in-difference procedures (Appendix Tables S16 and S17).

## Discussion

In this study using large, national data on SUD treatment use across two policy periods, we found that eviction ban expirations were associated with measurable increases in outpatient SUD care. State-level expirations in 2020 were linked to a 3% relative rise in the number of patients with an outpatient SUD visit and a 7% relative rise in patients with an outpatient OUD visit. The federal eviction moratorium in 2021 produced a 5% relative rise in patients with an outpatient SUD visit, 6% relative rise in patients with an outpatient OUD, and a 3% relative rise in patients with an SUD-related medication event.

Patients with inpatient stays changed little in either period. These patterns suggest that the re-emergence of eviction risk mainly altered use of lower acuity services delivered in office or clinic settings rather than more intensive hospital care.

Although several mechanisms could explain the direction and size of the effects, the most plausible in our view is that, when eviction filings resumed, stress and housing instability heightened substance use and symptoms, prompting more people to require and seek outpatient support.^36^ This increased demand for services appears to have superseded any disruptions in SUD care access associated with moratorium expiration. Receipt of a SUD related medication rose only after the federal ban ended, which could point to very different pandemic era social services available between Phase I and Phase II (2020 and 2021). Because our outpatient outcome combines in-person and telehealth visits, part of the observed increase could reflect shifts in visit mode as clinic operations and distancing practices changed, in addition to any change in underlying treatment need.

The absence of a clear effect on individuals with inpatient stays is noteworthy. Hospital admission often reflects acute medical need, crisis, or more intensive treatment needs, conditions that may be less sensitive to changes in rent enforcement over a 16-week window. However, we are unable to distinguish stays across different American Society of Addiction Medicine (ASAM) criteria^37^ and are only able to observe hospital-based SUD care. During this period, there was also a desire to keep people physically separate to prevent the spread of COVID, potentially blunting any inpatient effects, this also possibly explains why heightened symptoms and potential crises are leading to outpatient care (where people are more likely to be able to be physically distant) than inpatient care (which is also much less commonly used).^38^

Our results can be placed within a complex body of work on housing instability and substance use. Prior surveys have shown that renters behind on payments report higher levels of psychological distress^19,39,40^ and substance use,^41^ but evidence on treatment engagement is mixed. By using objective claims from nearly all U.S. pharmacies and 96% of physicians, our study adds population-level estimates and suggests potential increases in SUD outpatient treatment use. Our results also allow us to contextualize recent findings that expiring moratoria were associated with small, non-statistically significant increases in fatal overdoses.^42^ Increased outpatient SUD treatment following moratorium expiration may have acted as a countervailing force to eviction-associated stress and substance use, mitigating the impact of the expiration on overdoses.

### Limitations

This study has important limitations. First, SUD identification relies on diagnosis codes, and SUD may not be as well-documented in claims; we used ICD-10 algorithms and combined primary and secondary fields to improve capture. Second, 2020 and 2021 were dynamic policy environments and the fixed effects and control variables we use may not control for all possible confounders. Because early moratorium lifting often coincided with other reopening-related changes, our estimates may capture the combined effect of renewed eviction activity and changes in health care access, and we cannot fully separate these channels. Third, our 16-week post-expiration window (Phase 1) cannot assess longer-term outcomes, but it isolates the immediate period of heightened housing uncertainty when policy effects are likely largest. Fourth, event-study estimates for outpatient visits show lead coefficients that are close to zero and not statistically different from zero, but the pattern is consistent with a small pre-expiration divergence between treated and comparison states. If treated states were already increasing outpatient SUD care slightly faster before expiration, our DiD estimates may overstate the post-expiration increase. We therefore interpret outpatient effects cautiously and place more weight on the short-run window and complementary Phase 2 results. Fifth, the IQVIA data does not observe the entire continuum of SUD care (omits opioid treatment programs and residential treatment facilities). More broadly, IQVIA Dx reflects encounters submitted through participating professional billing systems and therefore covers a large share of insured care but not the full U.S. population. Coverage may differ across states and over time as payer participation and billing practices change, and the data are likely less complete for uninsured or cash-pay care and for services delivered in settings that do not submit standard claims. We do not have a state-by-state denominator of all patients receiving SUD care, so results should be interpreted as patterns among patients whose care is captured in these all-payer claims files. Further, inpatient data in the IQVIA data may not be as complete as the outpatient data, given the nature of open claims,^43^ limiting our interpretation of those findings. From March through July of 2020, there was a national moratorium on evictions under the CARES Act, limited to properties with federally-backed mortgages. Although we control for this with period fixed effects, these protections may have blunted the impact of expirations in Phase 1. Finally, there was substantial heterogeneity in protections afforded under state moratoria,^13^ with some states halting the entire eviction process, while others only banned enforcement.^15^ We did not explore potential effect modification in Phase 1 analyses. Similarly in Phase 2, there is evidence that not all states complied with the federal moratorium, which may have affected the pre-period in Phase 2 and potentially biased the results toward the null.^44^

## Conclusions

In sum, lifting eviction protections increased the rates of individuals obtaining outpatient SUD care: weekly counts of individuals with a clinic visit and medication events for SUDs a relative rise of 3%-6% after state bans ended and by 2%-5% after the federal ban lapsed, while individuals with an inpatient admission remained stable. These small but consistent changes suggest that threats to housing security can alter treatment seeking within weeks. Housing and health programs should therefore plan together. Policymakers weighing new measures to prevent or enable eviction should factor in these downstream effects on behavioral health systems and budget for outreach and treatment expansion when protections change.

## Supplementary Material

qxag006_Supplementary_Data
