# Supplementary material for: Lifting eviction moratoria shifted treatment for substance use disorders
Source: Health Aff Sch. 2026 Jan 10;4(2):qxag006. doi: 10.1093/haschl/qxag006 (PMC12898920; doi:10.1093/haschl/qxag006)

**APPENDIX**

**Appendix Table 1. SUD ICD Diagnosis Codes**

| ICD-10 Codes | Description |
| --- | --- |
| F10 | Alcohol related disorders |
| F11 | Opioid related disorders |
| F12 | Cannabis related disorders |
| F13 | Sedative, hypnotic, or anxiolytic related disorders |
| F14 | Cocaine related disorders |
| F15 | Other stimulant related disorders |
| F16 | Hallucinogen related disorders |
| F18 | Inhalant related disorders |
| F19 | Other psychoactive substance related disorders |

**Appendix Table 2. Places of Service Description**

| Type | Place of Service Description |
| --- | --- |
| Outpatient | office |
|  | hospital outpatient |
|  | non-residential substance abuse treatment facility |
|  | clinic - freestanding |
|  | on campus - outpatient hospital |
|  | community mental health center |
|  | independent clinic |
|  | clinic - community mental health center |
|  | special facility - other |
|  | federally qualified health center |
|  | off campus - outpatient hospital |
|  | clinic - other |
|  | clinic - federally qualified health center (FQHC) |
|  | psychiatric facility - partial hospitalization |
|  | public health clinic |
|  | clinic - comprehensive outpatient rehabilitation facility (CORF) |
|  | clinic - outpatient rehabilitation facility (ORF) |
|  | rural health clinic |
|  | comprehensive outpatient rehabilitation facility |
|  | clinic - rural health |
|  | school |
|  | mobile unit |
|  | tribal 638 free-standing facility |
|  | Indian health service free-standing facility |
|  | |
| Inpatient | inpatient hospital |
|  | hospital inpatient (including Medicare Part A) |
|  | inpatient psychiatric facility |
|  | comprehensive inpatient rehabilitation facility |
|  | hospital inpatient (Medicare Part B only) |

**Appendix Table 3. SUD Medication NDC Codes and Procedure Codes**

| Type | NDC Codes |
| --- | --- |
| Buprenorphine | 00054017613, 00054017713, 00054018813, 00054018913, 00093537856, 00093537956, 00093572056, 00093572156, 00143924605, 00228315303, 00228315403, 00228315473, 00228315503, 00228315567, 00228315573, 00228315603, 00378092393, 00378092493, 00378876716, 00378876793, 00378876816, 00378876893, 00406192303, 00406192403, 00406800503, 00406802003, 00490005100, 00490005130, 00490005160, 00490005190, 00781721606, 00781721664, 00781722706, 00781722764, 00781723806, 00781723864, 00781724906, 00781724964, 12496010001, 12496010002, 12496010005, 12496030001, 12496030002, 12496030005, 12496120201, 12496120203, 12496120401, 12496120403, 12496120801, 12496120803, 12496121201, 12496121203, 12496127802, 12496128302, 12496130602, 12496131002, 16590066605, 16590066630, 16590066705, 16590066730, 16590066790, 23490927003, 23490927006, 23490927009, 35356000407, 35356000430, 35356055530, 35356055630, 38779088800, 38779088801, 38779088803, 38779088805, 38779088806, 38779088809, 42291017430, 42291017530, 42858050103, 42858050203, 43063018407, 43063018430, 43063066706, 43063075306, 43598057901, 43598057930, 43598058001, 43598058030, 43598058101, 43598058130, 43598058201, 43598058230, 47781035503, 47781035511, 47781035603, 47781035611, 47781035703, 47781035711, 47781035803, 47781035811, 49452129201, 49452129202, 49452129203, 49452825301, 49452825302, 49452825303, 49452825304, 49999039507, 49999039515, 49999039530, 49999063830, 49999063930, 50090292400, 50268014411, 50268014415, 50268014511, 50268014515, 50383028793, 50383029493, 50383092493, 50383093093, 51552076501, 51552076502, 51552076505, 51552076506, 51552076509, 51552076510, 51552076550, 51927101200, 52427069203, 52427069211, 52427069403, 52427069411, 52427069803, 52427069811, 52427071203, 52427071211, 52440010014, 52959030430, 52959074930, 53217013830, 53217024630, 54123011430, 54123090730, 54123091430, 54123092930, 54123095730, 54123098630, 54569549600, 54569573900, 54569573901, 54569573902, 54569639900, 54569640800, 54569657800, 54868570700, 54868570701, 54868570702, 54868570703, 54868570704, 54868575000, 55045378403, 55700014730, 55700018430, 55700030230, 55700030330, 55700056804, 55887031204, 55887031215, 58118017608, 58118017708, 58118315608, 58284010014, 59385001201, 59385001230, 59385001401, 59385001430, 59385001601, 59385001630, 60429058611, 60429058630, 60429058633, 60429058711, 60429058730, 60429058733, 60687048111, 60687048121, 60687049211, 60687049221, 60846097003, 60846097103, 62175045232, 62175045832, 62756045964, 62756045983, 62756046064, 62756046083, 62756096964, 62756096983, 62756097083, 62991158301, 62991158302, 62991158303, 62991158304, 62991158306, 62991158307, 62991158308, 63275992201, 63275992202, 63275992203, 63275992204, 63275992205, 63275992207, 63370090506, 63370090509, 63370090510, 63370090515, 63481016101, 63481016160, 63481020701, 63481020760, 63481034801, 63481034860, 63481051901, 63481051960, 63481068501, 63481068560, 63481082001, 63481082060, 63481095201, 63481095260, 63629402801, 63629403401, 63629403402, 63629403403, 63629409201, 63629712501, 63629712502, 63629712503, 63629712504, 63629712505, 63629712506, 63629712507, 63629712601, 63629712602, 63629712603, 63629712604, 63629712605, 63629712606, 63629712607, 63629712608, 63629712609, 63629727001, 63629727002, 63874108403, 63874108503, 63874117303, 63874117403, 64725093003, 64725192403, 65162041503, 65162041509, 65162041603, 65162041609, 66336001530, 66336001630, 67046099030, 67046099130, 67046099230, 67046099330, 67046099430, 67046099530, 67046099630, 67046099730, 67046099830, 67046099930, 68071138003, 68071151003, 68258299103, 68258299903, 68308020230, 68308020830, 69189059101, 70518044200, 70518065200, 70518065201, 70518065202, 70518071100, 70518071101, 70518071102, 70518100700, 70518155700, 70518162500, 70518168400, 70518201400, 70518221600, 70518221700, 70518221800, 70518222600, 70518231100, 70518232700, 71335035301, 71335035302, 71335035303, 71335035304, 71335035305, 71335035306, 71335035307, 71335095001, 71335095002, 71335095003, 71335095004, 71335095005, 71335095006, 71335095007, 71335115401, 71335115402, 71335115403, 71335115404, 71335115405, 71335115406, 71335115407, 71335115408, 71335115409, 71335116301, 71335116302, 71335116303, 71335116304, 71335116305, 71335116306, 71335116307, 71335116308, 71335116309, 71335129601, 71335129602, 71335137801 |
| Naltrexone | *Oral*  00056001122, 00056001122, 00056001130, 00056001130, 00056001170, 00056001170, 00056007950, 00056007950, 00056008050, 00056008050, 00185003901, 00185003901, 00185003930, 00185003930, 00406009201, 00406009203, 00406117001, 00406117001, 00406117003, 00406117003, 00555090201, 00555090201, 00555090202, 00555090202, 10695006014, 10695006017, 16279008101, 16279008110, 16729008101, 16729008101, 16729008110, 16729008110, 38779088703, 38779088703, 38779088704, 38779088704, 38779088705, 38779088705, 38779088706, 38779088706, 38779088708, 38779088708, 38779088730, 38779088740, 38779088750, 38779088760, 42291063230, 42291063230, 43063059115, 43063059115, 47335032608, 47335032618, 47335032683, 47335032683, 47335032688, 47335032688, 49452480801, 49452480802, 49452483501, 49452483501, 49452483502, 49452483502, 49452483503, 49452483505, 50436010501, 50436010501, 51224020630, 51224020630, 51224020650, 51224020650, 51285027501, 51285027501, 51285027502, 51285027502, 51552073701, 51552073701, 51552073702, 51552073702, 51552073704, 51552073704, 51927275300, 51927275300, 51927354800, 51927354800, 51927360200, 51927360200, 51927437700, 51927437700, 52152010502, 52152010502, 52152010504, 52152010504, 52152010530, 52152010530, 52372075101, 52372075101, 52372075102, 52372075102, 52372075103, 52372075103, 53217026130, 54569672000, 54569913900, 54868557400, 54868557400, 55812033301, 55812033302, 55812033303, 58597840701, 58597840702, 58597840704, 58597840706, 60966014404, 60966024403, 60966034402, 62991124301, 62991124301, 62991124302, 62991124302, 62991124303, 62991124303, 62991124304, 62991124304, 62991312501, 62991312502, 62991312503, 62991312504, 63275990101, 63275990102, 63275990103, 63275990104, 63275990105, 63370015810, 63370015810, 63370015815, 63370015815, 63370015825, 63370015825, 63370015835, 63370015835, 63370015845, 63459030042, 65694010003, 65694010003, 65694010010, 65694010010, 65757030001, 65757030202, 68084029111, 68084029111, 68084029121, 68084029121, 68094085362, 68094085362, 68115068030, 68115068030, 70350782001, 71335001401, 71335001402, 71335001403, 76519116005  *Injectable*  00063450300, 00065757030, 00110980311, 63459030042, 65757030001 |
| Acamprosate | 00093535286, 00258400060, 00378633380, 00456333001, 00456333060, 00456333063, 10135063632, 42291010418, 51079024101, 51079024106, 54569576700, 54868529300, 55045329601, 60687012125, 60687012195, 68382056928, 68462043518 |
| Disulfiram | 00046080981, 00046081050, 00046081091, 00054035613, 00054035625, 00054035713, 00054035725, 00093503501, 00093503601, 00157066301, 00182053201, 00182053210, 00182053301, 00182053305, 00223071801, 00223071802, 00223071901, 00302231001, 00304080101, 00304080250, 00349846850, 00364033601, 00364033750, 00378414001, 00378414101, 00405436301, 00536376701, 00536376805, 00536376806, 00537600401, 00537600505, 00537600550, 00580119101, 00580157501, 00591536800, 00591536801, 00591536803, 00591537601, 00591537604, 00603343121, 00603343221, 00603343321, 00677100101, 00719132010, 00719132110, 00779037825, 00779037925, 00781106001, 00781107001, 00781107050, 00814262514, 00814262708, 00814262714, 00839128606, 00839128706, 00904118060, 00904118061, 00904118151, 00904118160, 17022390302, 35470012801, 38779197102, 38779197105, 38779197108, 38779197109, 47202247201, 47202248501, 47781060730, 49452264501, 49452264502, 49452264503, 49884015301, 49884015305, 49884015401, 49884015403, 49884015405, 50111033101, 50111033103, 50111033201, 50111033202, 51285052302, 51285052402, 51552109505, 51655023224, 51728053801, 51728053901, 51927312100, 52406061001, 54569179002, 54868503400, 54868503401, 54868503402, 58469037673, 58469037683, 58469376730, 58469376830, 60429019601, 60429019630, 62991159401, 62991159402, 64980017101, 64980017103, 64980017201, 64980017203, 65473070601, 65473070701 |

**Appendix Table 4. SUD Medication Procedure Codes**

| Type | Procedure Code | Description |
| --- | --- | --- |
| Oral buprenorphine/naloxone, buprenorphine | H0033 (if dx=OUD) | Oral medical administration, direct observation |
|  | J0571 | buprenorphine/naloxone, oral |
|  | J0572 | buprenorphine/naloxone, oral |
|  | J0573 | buprenorphine/naloxone, oral |
|  | J0574 | buprenorphine/naloxone, oral |
|  | J0575 | buprenorphine/naloxone, oral |
|  | 96372 (if dx=OUD/AUD) | Intramuscular/subcutaneous injection |
|  | G2068 (after 1/1/2020) | Medication assisted treatment, buprenorphine (oral); weekly bundle for OTPs |
|  | G2079 (after 1/1/2020) | Take-home supply of buprenorphine (oral) for OTPs; list separately in addition to code for primary procedure. |
| Buprenorphine Long Acting (injection) | Q9991 (after 7/1/2018) | Injection, buprenorphine extended-release, less than or equal to 100 mg |
|  | Q9992 (after 7/1/2018) | Injection, buprenorphine extended-release, greater than 100 mg |
|  | G2069 (after 1/1/2020) | Medication assisted treatment, buprenorphine (injectable); weekly bundle for OTPs. |
| Buprenorphine Long Acting (implantation) | 11981 (if dx1=OUD) | Insertion, non-biodegradable drug delivery implant |
|  | 11983 (if dx1=OUD) | Removal with reinsertion, non-biodegradable drug delivery implant |
|  | G0516 (if dx1=OUD) | Insert drug delivery implant, ≥4 |
|  | G0518 (if dx1=OUD) | Removal with reinsertion, non-biodegradable drug delivery implants, ≥4 |
|  | G2070 (after 1/1/2020) | Medication assisted treatment, buprenorphine (implant insertion); weekly bundle for OTPs. |
|  | G2072 (after 1/1/2020) | Medication assisted treatment, buprenorphine (implant insertion and removal); weekly bundle for OTPs. |
|  | J0570 (after 1/1/2017) | buprenorphine, implant 74.2mg |
| Oral methadone | H0020 | Alcohol and/or drug services; methadone administration and/or service |
|  | S0109 | Oral administration of methadone |
|  | H0033 (if dx=OUD) | Oral medical administration, direct observation |
|  | G2078 (after 1/1/2020) | Take-home supply of methadone for OTPs; up to 7 additional day supply; list separately in addition to code for primary procedure |
|  | G2067 (after 1/1/2020) | Medication assisted treatment, methadone; weekly bundle for OTPs |
| Methadone injection | J1230 | Methadone injection |
| Methadone Maintenance | HZ81ZZZ | Medication management for substance abuse treatment, methadone maintenance (ICD-10 code) |
|  | HZ91ZZZ | Pharmacotherapy for substance abuse treatment, methadone maintenance (ICD-10 code) |
| Extended-release injectable naltrexone for OUD treatment (and AUD treatment) (Vivitrol) | J2315 | Injection, naltrexone, depot form, 1 mg |

**Appendix Table 5. Unweighted Summary Statistics (Mean Patient Counts by State-Week)**

| **Panel A. Phase 1 (March 9^th^–August 30^th^, 2020)** | | | | | | | | | | | | |
| --- | --- | --- | --- | --- | --- | --- | --- | --- | --- | --- | --- | --- |
|  | **Treatment (N of states = 26)** | | | | | | **Control (N of states = 18)** | | | | | |
|  | **State Moratorium ON** | | | **State Moratorium OFF** | | | **Always moratorium** | | |  | | |
|  | **N** | **Mean** | **SD** | **N** | **Mean** | **SD** | **N** | **Mean** | **SD** | **N** | **Mean** | **SD** |
| **SUD Outpatient** | 268 | 1,028.14 | 809.20 | 274 | 998.04 | 801.08 | 437 | 2,419.14 | 2,002.41 |  |  |  |
| **SUD Inpatient** | 268 | 169.57 | 135.51 | 274 | 167.32 | 149.88 | 437 | 389.63 | 338.61 |  |  |  |
| **SUD Medication** | 268 | 2,609.37 | 2,008.57 | 274 | 2,285.85 | 1,870.91 | 437 | 5,634.36 | 4,250.18 |  |  |  |
| **OUD Outpatient** | 268 | 450.06 | 444.48 | 274 | 411.32 | 423.12 | 437 | 877.66 | 759.02 |  |  |  |
| **OUD Inpatient** | 268 | 24.04 | 22.74 | 274 | 21.56 | 20.00 | 437 | 58.58 | 56.18 |  |  |  |

| **Panel B. Phase 2 (June 7^th^–December 26^th^, 2021)** | | | | | | | | | | | | |
| --- | --- | --- | --- | --- | --- | --- | --- | --- | --- | --- | --- | --- |
|  | **Treatment (N of states = 42)** | | | | | | **Control (N of states = 9)** | | | | | |
|  | **Federal Moratorium ON** | | | **Federal and State Moratorium OFF** | | | **Always Moratorium** | | | **Federal Moratorium OFF/State Moratorium ON** | | |
|  | **N** | **Mean** | **SD** | **N** | **Mean** | **SD** | **N** | **Mean** | **SD** | **N** | **Mean** | **SD** |
| **SUD Outpatient** | 462 | 1,504.24 | 1,807.24 | 756 | 1,498.53 | 1,716.98 | 99 | 3,628.52 | 2,472.51 | 142 | 3,565.08 | 2,516.16 |
| **SUD Inpatient** | 462 | 211.30 | 200.45 | 756 | 191.51 | 179.39 | 99 | 580.72 | 427.46 | 142 | 540.73 | 408.73 |
| **SUD Medication** | 462 | 3,386.15 | 3,106.87 | 756 | 3,506.40 | 3,189.07 | 99 | 7,502.46 | 5,126.25 | 142 | 7,432.19 | 5,455.59 |
| **OUD Outpatient** | 462 | 623.34 | 948.65 | 756 | 630.98 | 907.09 | 99 | 1,304.72 | 947.89 | 142 | 1,306.49 | 975.70 |
| **OUD Inpatient** | 462 | 30.72 | 33.01 | 756 | 28.00 | 29.80 | 99 | 92.82 | 69.76 | 142 | 86.58 | 70.05 |

*Notes:* N’s refer to the number of state-weeks. In the Phase 1 analysis, the treatment is the expiration of state-level eviction moratorium expiration of states in the treatment group. States in the control group include those that had state-level moratoriums but their moratorium did not expire during our observation period. In the Stage 2 analysis, the treatment is the expiration of federal-level eviction moratorium expiration. States in the control group include those that continued to have state-level moratoriums after the expiration of the federal moratorium.

**Appendix Table 6. Weighted Summary Statistics (mean patient counts by state-week)**

| Panel A. Phase 1 | | | | | | | | | | | | |
| --- | --- | --- | --- | --- | --- | --- | --- | --- | --- | --- | --- | --- |
|  | Treatment (N of states = 26) | | | | | | Control (N of states = 18) | | | | | |
|  | Moratorium ON | | | Moratorium OFF | | | Always moratorium | | |  | | |
|  | N | Mean | SD | N | Mean | SD | N | Mean | SD | N | Mean | SD |
| SUD Outpatient | 268 | 1,521.95 | 786.23 | 274 | 1,572.70 | 862.26 | 437 | 3,900.42 | 2,157.93 |  |  |  |
| SUD Inpatient | 268 | 293.98 | 174.69 | 274 | 333.63 | 230.77 | 437 | 667.93 | 359.05 |  |  |  |
| SUD Medication | 268 | 3,471.72 | 1,736.15 | 274 | 3,294.88 | 1,676.29 | 437 | 8,482.69 | 3,656.02 |  |  |  |
| OUD Outpatient | 268 | 593.96 | 432.87 | 274 | 580.04 | 434.81 | 437 | 1,341.51 | 784.31 |  |  |  |
| OUD Inpatient | 268 | 34.81 | 21.41 | 274 | 35.89 | 22.27 | 437 | 88.30 | 48.66 |  |  |  |

| Panel B. Phase 2 | | | | | | | | | | | | |
| --- | --- | --- | --- | --- | --- | --- | --- | --- | --- | --- | --- | --- |
|  | Treatment (N of states = 42) | | | | | | Control (N of states = 9) | | | | | |
|  | Moratorium ON | | | Moratorium OFF | | | Moratorium ON | | | Moratorium OFF | | |
|  | N | Mean | SD | N | Mean | SD | N | Mean | SD | N | Mean | SD |
| SUD Outpatient | 462 | 2,347.52 | 2,318.14 | 756 | 2,330.61 | 2,167.43 | 99 | 5,299.46 | 2,105.98 | 142 | 5402.19 | 1983.47 |
| SUD Inpatient | 462 | 382.43 | 262.67 | 756 | 345.60 | 241.48 | 99 | 905.74 | 385.51 | 142 | 869.24 | 344.76 |
| SUD Medication | 462 | 5,171.94 | 3,516.46 | 756 | 5,370.47 | 3,692.31 | 99 | 10,323.58 | 3,481.55 | 142 | 10,642.56 | 3,650.41 |
| OUD Outpatient | 462 | 967.14 | 1,251.51 | 756 | 973.13 | 1,174.66 | 99 | 1,771.07 | 861.25 | 142 | 1,829.15 | 820.30 |
| OUD Inpatient | 462 | 51.47 | 38.48 | 756 | 46.42 | 36.87 | 99 | 119.40 | 51.29 | 142 | 113.79 | 51.45 |

*Notes:* In the Stage 1 analysis, the treatment is the expiration of state-level eviction moratorium expiration of states in the treatment group. States in the control group include those that had state-level moratoriums but their moratorium did not expire during our observation period. In the Stage 2 analysis, the treatment is the expiration of federal-level eviction moratorium expiration. States in the control group include those that continued to have state-level moratoriums after the expiration of the federal moratorium.

**Appendix Table 7. Percent Change in Outcomes Attributable to Eviction Moratorium Expiration during Phase 2, excluding state-weeks with more than 10 % of the state population under a local (city or county) moratorium**

|  | **N** | **Pre-period (mean)** | **Post-period (mean)** | **Percent Change**  **(95% CI)** | **P-value** | **Percent Change**  **(95% CI) (Graphical)** |
| --- | --- | --- | --- | --- | --- | --- |
| **SUD outpatient** | | | | | | 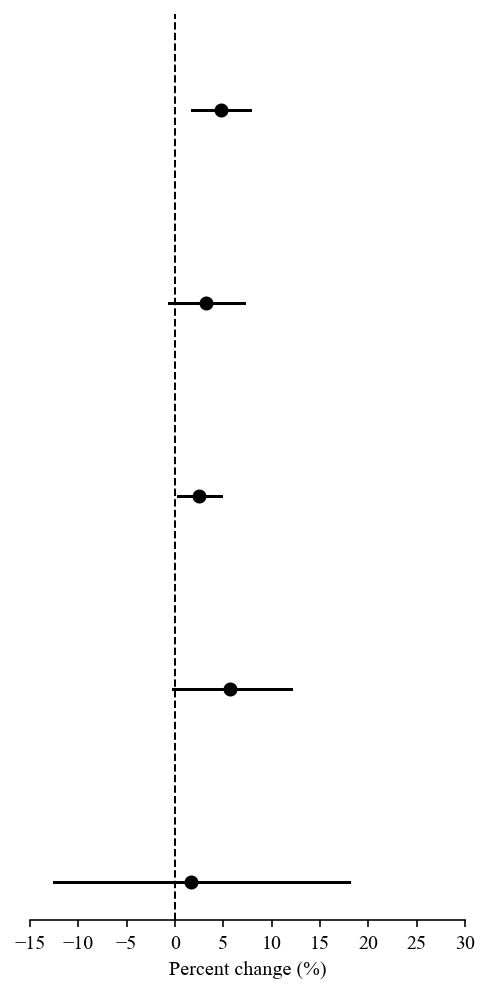 |
| Treated States | 42 | 1504 | 1499 | 4.76%  (1.80%, 7.72%) | 0.002 |  |
| Untreated States | 9 | 3629 | 3565 |  |  |  |
| **SUD inpatient** | | | | | |  |
| Treated States | 42 | 211 | 192 | 3.15%  (-0.65%, 7.09%) | 0.106 |  |
| Untreated States | 9 | 581 | 541 |  |  |  |
| **SUD medication** | | | | | |  |
| Treated States | 42 | 3386 | 3506 | 2.49%  (0.27%, 4.75%) | 0.028 |  |
| Untreated States | 9 | 7502 | 7432 |  |  |  |
| **OUD outpatient** | | | | | |  |
| Treated States | 42 | 623 | 631 | 5.73%  (-0.20%, 12.02%) | 0.059 |  |
| Untreated States | 9 | 1305 | 1306 |  |  |  |
| **OUD inpatient** | | | | | |  |
| Treated States | 42 | 31 | 28 | 1.61%  (-12.54%, 18.01%) | 0.834 |  |
| Untreated States | 9 | 93 | 87 |  |  |  |

***Notes:*** In the Phase 2 analysis, a standard difference-in-difference method is used for analysis. The Poisson model is used for estimation. The treatment is the expiration of the federal eviction moratorium policy on August 26, 2021. The data sample includes 42 states in the treatment group and 9 states in the control group, with IL and WA censored periods after state-level eviction moratorium expiration. The study period is from week 23 to week 51 in 2021. States in the control group include those that continued to have state-level moratoriums after the expiration of the federal moratorium. The number of observations is 1,459 for each. State and year-month fixed effects are included. VT in September, November and December has >10% population covered by a local (city or county) moratorium. WA in November and December has >10% population covered by a local (city or county) moratorium. We exclude any state-weeks within this range for the sensitivity study.

**Appendix Table 8. Percent Change in Outcomes Attributable to Eviction Moratorium Expiration during Phase 1, Adding COVID Policy Stringency Measure as an Additional Control Variable**

|  | **N** | **Pre-period (mean)** | **Post-period (mean)** | **Percent Change**  **(95% CI)** | **P-value** | **Percent Change**  **(95% CI) (Graphical)** |
| --- | --- | --- | --- | --- | --- | --- |
| **SUD outpatient** | | | | | | 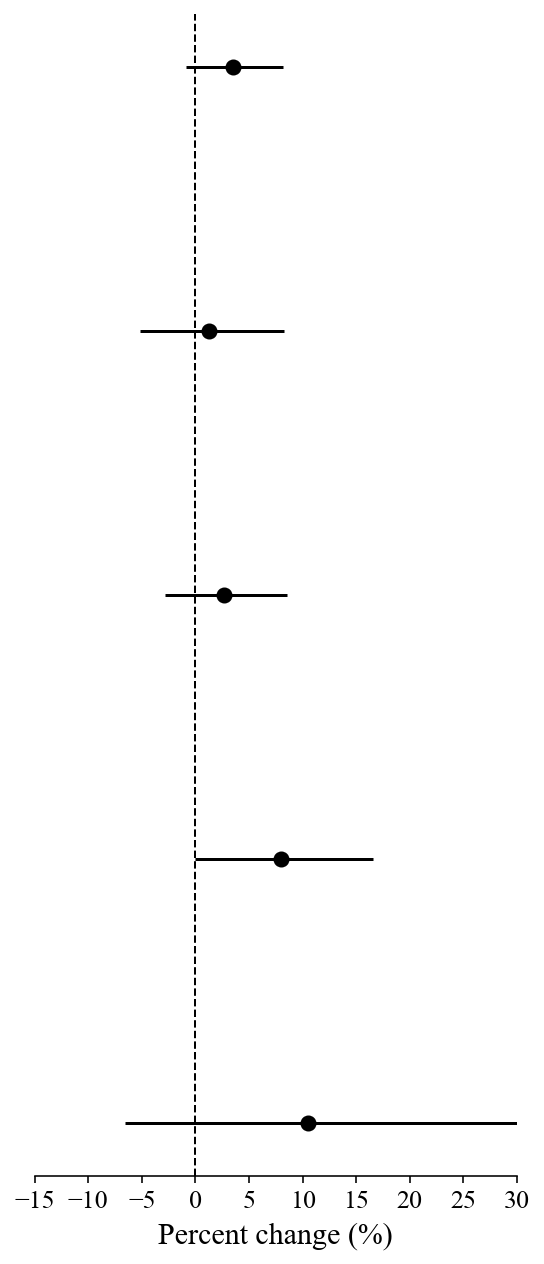 |
| Treated States | 26 | 1028 | 998 | 3.53%  (-0.88%, 8.13%) | 0.119 |  |
| Untreated States | 18 | 2419 | |  |  |  |
| **SUD inpatient** | | | | | |  |
| Treated States | 26 | 170 | 167 | 1.31%  (-5.19%, 8.25%) | 0.701 |  |
| Untreated States | 18 | 390 | |  |  |  |
| **SUD medication** | | | | | |  |
| Treated States | 26 | 2609 | 2286 | 2.69%  (-2.86%, 8.56%) | 0.349 |  |
| Untreated States | 18 | 5634 | |  |  |  |
| **OUD outpatient** | | | | | |  |
| Treated States | 26 | 450 | 411 | 7.95%  (-0.03%, 16.56%) | 0.051 |  |
| Untreated States | 18 | 878 | |  |  |  |
| **OUD inpatient** | | | | | |  |
| Treated States | 26 | 24 | 22 | 10.47%  (-6.58%, 30.64%) | 0.244 |  |
| Untreated States | 18 | 59 | |  |  |  |

***Notes:*** In the Phase 1 analysis, Callaway & Sant’ Anna’s estimation method is used for analysis. The log version of the three outcomes is taken for estimation. The treatment is the expiration of the state-level eviction moratorium policy. The data sample includes 44 states having state-level eviction moratoriums. The study period is from week 11 to week 35 in 2020, starting from the week when the state’s eviction moratorium started. The treatment is the expiration of state-level eviction moratorium expiration of states in the treatment group. States in the control group include those that had state-level moratoriums but their moratorium did not expire during our observation period. The number of observations is 979 for each.

**Appendix Table 9. Percent Change in Outcomes Attributable to Eviction Moratorium Expiration during Phase 2, Adding COVID Policy Stringency Measure as an Additional Control Variable**

|  | **N** | **Pre-period (mean)** | **Post-period (mean)** | **Percent Change**  **(95% CI)** | **P-value** | **Percent Change**  **(95% CI) (Graphical)** |
| --- | --- | --- | --- | --- | --- | --- |
| **SUD outpatient** | | | | | | 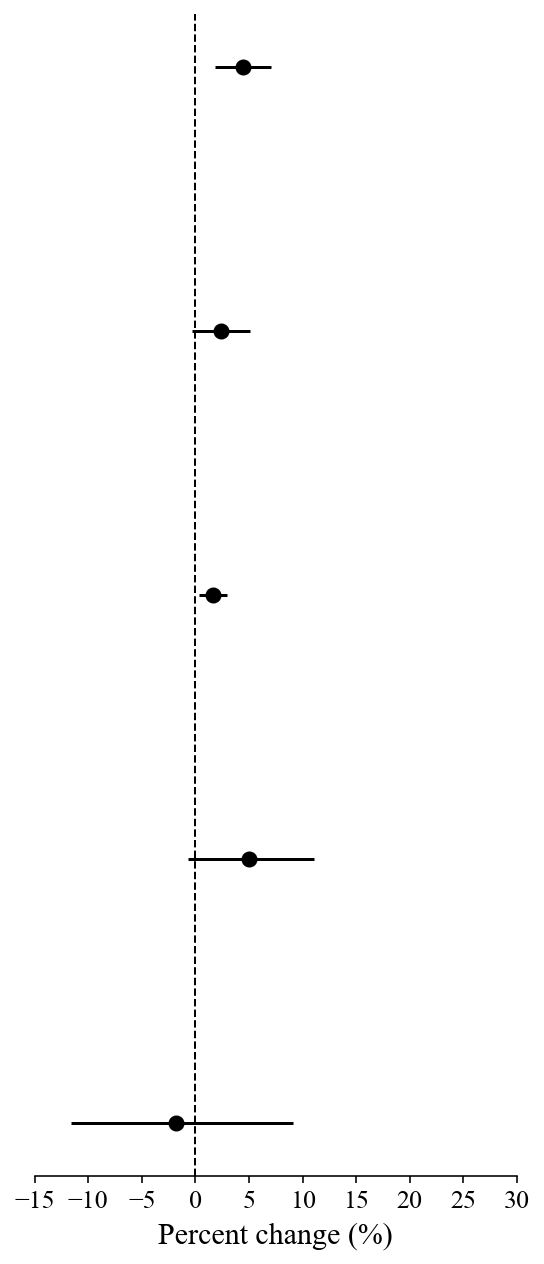 |
| Treated States | 42 | 1504 | 1499 | 4.40%  (1.82%, 7.05%) | 0.001 |  |
| Untreated States | 9 | 3629 | 3565 |  |  |  |
| **SUD inpatient** | | | | | |  |
| Treated States | 42 | 211 | 192 | 2.34%  (-0.35%, 5.10%) | 0.088 |  |
| Untreated States | 9 | 581 | 541 |  |  |  |
| **SUD medication** | | | | | |  |
| Treated States | 42 | 3386 | 3506 | 1.63%  (0.35%, 2.91%) | 0.012 |  |
| Untreated States | 9 | 7502 | 7432 |  |  |  |
| **OUD outpatient** | | | | | |  |
| Treated States | 42 | 623 | 631 | 5.04%  (-0.67%, 11.07%) | 0.085 |  |
| Untreated States | 9 | 1305 | 1306 |  |  |  |
| **OUD inpatient** | | | | | |  |
| Treated States | 42 | 31 | 28 | -1.79%  (-11.57%, 9.06%) | 0.736 |  |
| Untreated States | 9 | 93 | 87 |  |  |  |

***Notes:*** In the Phase 2 analysis, a standard difference-in-difference method is used for analysis. The Poisson model is used for estimation. The treatment is the expiration of the federal eviction moratorium policy on August 26, 2021. The data sample includes 42 states in the treatment group and 9 states in the control group, with IL and WA censored periods after state-level eviction moratorium expiration. The study period is from week 23 to week 51 in 2021. States in the control group include those that continued to have state-level moratoriums after the expiration of the federal moratorium. The number of observations is 1,459 for each. State and year-month fixed effects are included.

**Appendix Table 10. Percent Change in Outcomes Attributable to Eviction Moratorium Expiration, Removing Control Variables**

|  | **Estimates from the main model** | | **Removing SNAP, unemployment insurance** | | **Removing SNAP, unemployment insurance, ERA** | |
| --- | --- | --- | --- | --- | --- | --- |
|  | **Percentage Change**  **(95% CI)** | **P-value** | **Percentage Change**  **(95% CI)** | **P-value** | **Percentage Change**  **(95% CI)** | **P-value** |
| **Phase 1** | | | | | | |
| SUD outpatient | 3.28%  (-0.50%, 7.20%) | 0.090 | 3.18%  (-0.48%, 6.99%) | 0.090 | 3.18%  (-0.48%, 6.99%) | 0.090 |
| SUD inpatient | -1.10%  (-6.49%, 4.61%) | 0.699 | -1.27%  (-7.36%, 5.23%) | 0.695 | -1.27%  (-7.36%, 5.23%) | 0.695 |
| SUD medication | 0.58%  (-1.97%, 3.19%) | 0.659 | 0.63%  (-2.34%, 3.71%) | 0.680 | 0.63%  (-2.34%, 3.71%) | 0.680 |
| OUD outpatient | 6.72%  (0.29%, 13.59%) | 0.040 | 6.48%  (-0.11%, 13.51%) | 0.054 | 6.48%  (-0.11%, 13.51%) | 0.054 |
| OUD inpatient | 6.56%  (-9.37%, 25.26%) | 0.442 | 7.06%  (-8.83%, 25.72%) | 0.406 | 7.06%  (-8.83%, 25.72%) | 0.406 |
| **Phase 2** | | | | | | |
| SUD outpatient | 4.87%  (1.81%, 8.02%) | 0.002 | 5.16%  (2.00%, 8.43%) | 0.001 | 3.08%  (-1.32%, 7.68%) | 0.173 |
| SUD inpatient | 3.15%  (-0.65%, 7.09%) | 0.106 | 3.41%  (-0.49%, 7.46%) | 0.087 | 0.38%  (-2.75%, 3.62%) | 0.813 |
| SUD medication | 2.48%  (0.27%, 4.74%) | 0.028 | 2.76%  (0.35%, 5.24%) | 0.025 | 2.39%  (0.14%, 4.69%) | 0.037 |
| OUD outpatient | 5.72%  (-0.21%, 12.01%) | 0.059 | 5.74%  (-0.12%, 11.95%) | 0.055 | 3.37%  (-3.30%, 10.50%) | 0.330 |
| OUD inpatient | 1.61%  (-12.54%, 18.01%) | 0.834 | 1.78%  (-12.50%, 18.40%) | 0.819 | -0.18%  (-10.63%, 11.49%) | 0.974 |

***Notes:*** In the Phase 1 analysis, Callaway & Sant’ Anna’s estimation method is used for analysis. The log version of the three outcomes is taken for estimation. The treatment is the expiration of the state-level eviction moratorium policy. The data sample includes 44 states having state-level eviction moratoriums. The study period is from week 11 to week 35 in 2020, starting from the week when the state’s eviction moratorium started. The treatment is the expiration of state-level eviction moratorium expiration of states in the treatment group. States in the control group include those that had state-level moratoriums but their moratorium did not expire during our observation period. The number of observations is 979 for each. In the Phase 2 analysis, a standard difference-in-difference method is used for analysis. The Poisson model is used for estimation. The treatment is the expiration of the federal eviction moratorium policy on August 26, 2021. The data sample includes 42 states in the treatment group and 9 states in the control group, with IL and WA censored periods after state-level eviction moratorium expiration. The study period is from week 23 to week 51 in 2021. States in the control group include those that continued to have state-level moratoriums after the expiration of the federal moratorium. The number of observations is 1,459 for each. State and year-month fixed effects are included. Emergency rental assistance only affects Phase 2.

**Appendix Table 11. Percent Difference in Insurance Type Attributable to Eviction Moratorium Expiration**

| Claim sample of each of the following: | Share of commercial | | Share of Medicaid | |
| --- | --- | --- | --- | --- |
|  | **Coefficient**  **(95% CI)** | **P-value** | **Coefficient**  **(95% CI)** | **P-value** |
| Phase 1 | | | | |
| SUD outpatient | -0.0037  (-0.0103, 0.0029) | 0.267 | -0.0001  (-0.0105, 0.0103) | 0.984 |
| SUD inpatient | -0.0165  (-0.0403, 0.0074) | 0.176 | 0.0042  (-0.0138, 0.0223) | 0.646 |
| SUD medication | 0.0171  (-0.0076, 0.0418) | 0.176 | -0.0081  (-0.0327, 0.0164) | 0.517 |
| OUD outpatient | 0.0108  (-0.0066, 0.0283) | 0.225 | -0.0061  (-0.0237, 0.0114) | 0.494 |
| OUD inpatient | -0.0776  (-0.1445, -0.0106) | 0.023 | 0.0298  (-0.0205, 0.0801) | 0.245 |
| Phase 2 | | | | |
| SUD outpatient | 0.0019  (-0.0912, 0.0950) | 0.968 | -0.0152  (-0.0570, 0.0266) | 0.475 |
| SUD inpatient | 0.0335  (-0.1259, 0.1397) | 0.095 | -0.0191  (-0.0749, 0.0367) | 0.503 |
| SUD medication | 0.0501  (-0.0745, 0.1747) | 0.430 | -0.0745  (-0.1945, 0.0454) | 0.223 |
| OUD outpatient | 0.0253  (-0.1126, 0.1632) | 0.719 | -0.0086  (-0.0760, 0.0587) | 0.801 |
| OUD inpatient | 0.0421  (-0.0810, 0.1651) | 0.503 | 0.0205  (-0.0835, 0.1246) | 0.700 |

***Notes:*** In the Phase 1 analysis, Callaway & Sant’ Anna’s estimation method is used for analysis. The treatment is the expiration of the state-level eviction moratorium policy. The data sample includes 44 states having state-level eviction moratoriums. The study period is from week 11 to week 35 in 2020, starting from the week when the state’s eviction moratorium started. The treatment is the expiration of state-level eviction moratorium expiration of states in the treatment group. States in the control group include those that had state-level moratoriums but their moratorium did not expire during our observation period. The number of observations is 979 for each. In the Phase 2 analysis, a standard difference-in-difference method is used for analysis. The treatment is the expiration of the federal eviction moratorium policy on August 26, 2021. The data sample includes 42 states in the treatment group and 9 states in the control group, with IL and WA censored periods after state-level eviction moratorium expiration. The study period is from week 23 to week 51 in 2021. States in the control group include those that continued to have state-level moratoriums after the expiration of the federal moratorium. The number of observations is 1,459 for each. State and year-month fixed effects are included.

**Appendix Table 12. Percent Difference in Insurance Type Attributable to Eviction Moratorium Expiration**

| **Unique patient sample with any of the following:** | **Average age** | | **Share of female** | |
| --- | --- | --- | --- | --- |
|  | **Coefficient**  **(95% CI)** | **P-value** | **Coefficient**  **(95% CI)** | **P-value** |
| **Phase 1** | | | | |
| SUD outpatient | -0.0967  (-0.5949, 0.4016) | 0.704 | 0.0017  (-0.0064, 0.0098) | 0.678 |
| SUD inpatient | 1.0418  (-0.1874, 2.2710) | 0.097 | -0.0103  (-0.0258, 0.0052) | 0.193 |
| SUD medication | -0.1851  (-0.4025, 0.0324) | 0.095 | -0.0004  (-0.0055, 0.0047) | 0.875 |
| OUD outpatient | -0.1302  (-0.8662, 0.6058) | 0.729 | 0.0148  (-0.0012, 0.0309) | 0.071 |
| OUD inpatient | -0.3530  (-2.8502, 2.1443) | 0.782 | 0.0312  (-0.0346, 0.0969) | 0.353 |
| **Phase 2** | | | | |
| SUD outpatient | -0.0065  (-0.0162, 0.0032) | 0.191 | 0.0023  (-0.0118, 0.0164) | 0.749 |
| SUD inpatient | -0.0094  (-0.0170, -0.0017) | 0.016 | 0.0004  (-0.0317, 0.0324) | 0.982 |
| SUD medication | -0.0006  (-0.0027, 0.0015) | 0.592 | 0.0028  (-0.0022, 0.0079) | 0.273 |
| OUD outpatient | -0.0024  (-0.0098, 0.0049) | 0.518 | 0.0151  (0.0001, 0.0301) | 0.048 |
| OUD inpatient | -0.0092  (-0.0254, 0.0070) | 0.267 | -0.0469  (-0.1078, 0.0141) | 0.132 |

***Notes:*** In the Phase 1 analysis, Callaway & Sant’ Anna’s estimation method is used for analysis. The treatment is the expiration of the state-level eviction moratorium policy. The data sample includes 44 states having state-level eviction moratoriums. The study period is from week 11 to week 35 in 2020, starting from the week when the state’s eviction moratorium started. The treatment is the expiration of state-level eviction moratorium expiration of states in the treatment group. States in the control group include those that had state-level moratoriums but their moratorium did not expire during our observation period. The number of observations is 979 for each. In the Phase 2 analysis, a standard difference-in-difference method is used for analysis. The treatment is the expiration of the federal eviction moratorium policy on August 26, 2021. The data sample includes 42 states in the treatment group and 9 states in the control group, with IL and WA censored periods after state-level eviction moratorium expiration. The study period is from week 23 to week 51 in 2021. States in the control group include those that continued to have state-level moratoriums after the expiration of the federal moratorium. The number of observations is 1,459 for each. State and year-month fixed effects are included.

**Appendix Table 13. Percent Change in Outcomes Attributable to Eviction Moratorium Expiration during Phase 1, Unweighted by Population**

|  | **N** | **Pre-period (mean)** | **Post-period (mean)** | **Percent Change**  **(95% CI)** | **P-value** | **Percent Change**  **(95% CI) (Graphical)** |
| --- | --- | --- | --- | --- | --- | --- |
| **SUD outpatient** | | | | | | 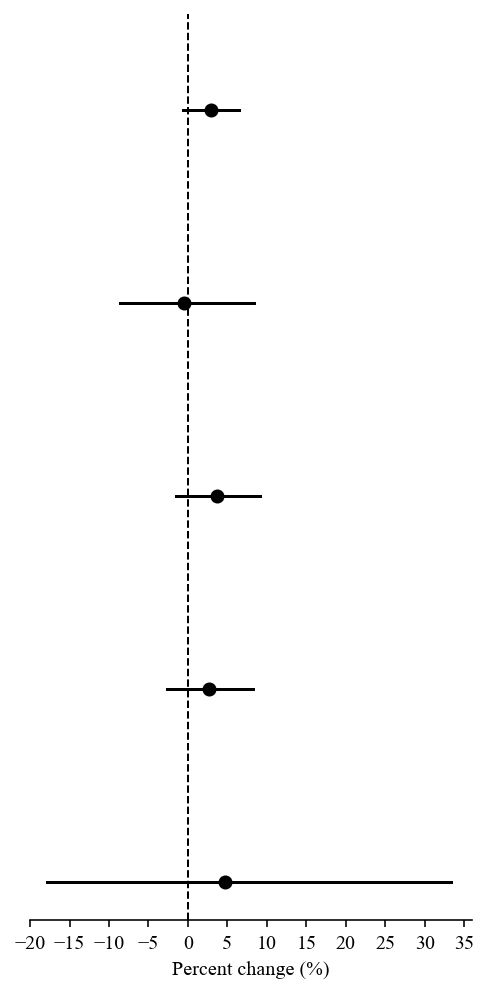 |
| Treated States | 26 | 1028 | 998 | 2.87%  (-0.59%, 6.45%) | 0.105 |  |
| Untreated States | 18 | 2419 | |  |  |  |
| **SUD inpatient** | | | | | |  |
| Treated States | 26 | 170 | 167 | -0.48%  (-8.59%, 8.35%) | 0.912 |  |
| Untreated States | 18 | 390 | |  |  |  |
| **SUD medication** | | | | | |  |
| Treated States | 26 | 2609 | 2286 | 3.66%  (-1.55%, 9.13%) | 0.172 |  |
| Untreated States | 18 | 5634 | |  |  |  |
| **OUD outpatient** | | | | | |  |
| Treated States | 26 | 450 | 411 | 2.64%  (-2.68%, 8.25%) | 0.338 |  |
| Untreated States | 18 | 878 | |  |  |  |
| **OUD inpatient** | | | | | |  |
| Treated States | 26 | 24 | 22 | 4.68%  (-17.83%, 33.31%) | 0.711 |  |
| Untreated States | 18 | 59 | |  |  |  |

***Notes:*** In the Phase 1 analysis, Callaway & Sant’ Anna’s estimation method is used for analysis. The log version of the three outcomes is taken for estimation. The treatment is the expiration of the state-level eviction moratorium policy. The data sample includes 44 states having state-level eviction moratoriums. The study period is from week 11 to week 35 in 2020, starting from the week when the state’s eviction moratorium started. The treatment is the expiration of state-level eviction moratorium expiration of states in the treatment group. States in the control group include those that had state-level moratoriums but their moratorium did not expire during our observation period. The number of observations is 979 for each.

**Appendix Table 14. Percent Change in Outcomes Attributable to Eviction Moratorium Expiration during Phase 2, Unweighted by Population**

|  | **N** | **Pre-period (mean)** | **Post-period (mean)** | **Percent Change**  **(95% CI)** | **P-value** | **Percent Change**  **(95% CI) (Graphical)** |
| --- | --- | --- | --- | --- | --- | --- |
| **SUD outpatient** | | | | | | 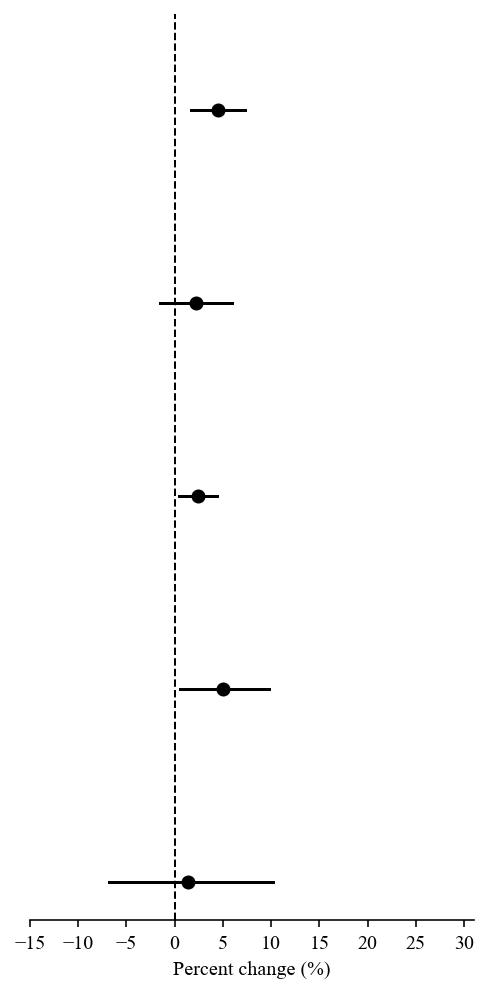 |
| Treated States | 42 | 1504 | 1499 | 4.48%  (1.72%, 7.33%) | 0.001 |  |
| Untreated States | 9 | 3629 | 3565 |  |  |  |
| **SUD inpatient** | | | | | |  |
| Treated States | 42 | 211 | 192 | 2.19%  (-1.48%, 5.99%) | 0.246 |  |
| Untreated States | 9 | 581 | 541 |  |  |  |
| **SUD medication** | | | | | |  |
| Treated States | 42 | 3386 | 3506 | 2.39%  (0.43%, 4.38%) | 0.016 |  |
| Untreated States | 9 | 7502 | 7432 |  |  |  |
| **OUD outpatient** | | | | | |  |
| Treated States | 42 | 623 | 631 | 5.02%  (0.50%, 9.74%) | 0.029 |  |
| Untreated States | 9 | 1305 | 1306 |  |  |  |
| **OUD inpatient** | | | | | |  |
| Treated States | 42 | 31 | 28 | 1.33%  (-6.79%, 10.15%) | 0.757 |  |
| Untreated States | 9 | 93 | 87 |  |  |  |

***Notes:*** In the Phase 2 analysis, a standard difference-in-difference method is used for analysis. The Poisson model is used for estimation. The treatment is the expiration of the federal eviction moratorium policy on August 26, 2021. The data sample includes 42 states in the treatment group and 9 states in the control group, with IL and WA censored periods after state-level eviction moratorium expiration. The study period is from week 23 to week 51 in 2021. States in the control group include those that continued to have state-level moratoriums after the expiration of the federal moratorium. The number of observations is 1,459 for each. State and year-month fixed effects are included.

**Appendix Table 15. Percent Change in Outcomes Attributable to Eviction Moratorium Expiration during Phase 2, Using Negative Binomial Model**

|  | **N** | **Pre-period (mean)** | **Post-period (mean)** | **Percent Change**  **(95% CI)** | **P-value** | **Percent Change**  **(95% CI) (Graphical)** |
| --- | --- | --- | --- | --- | --- | --- |
| **SUD outpatient** | | | | | | 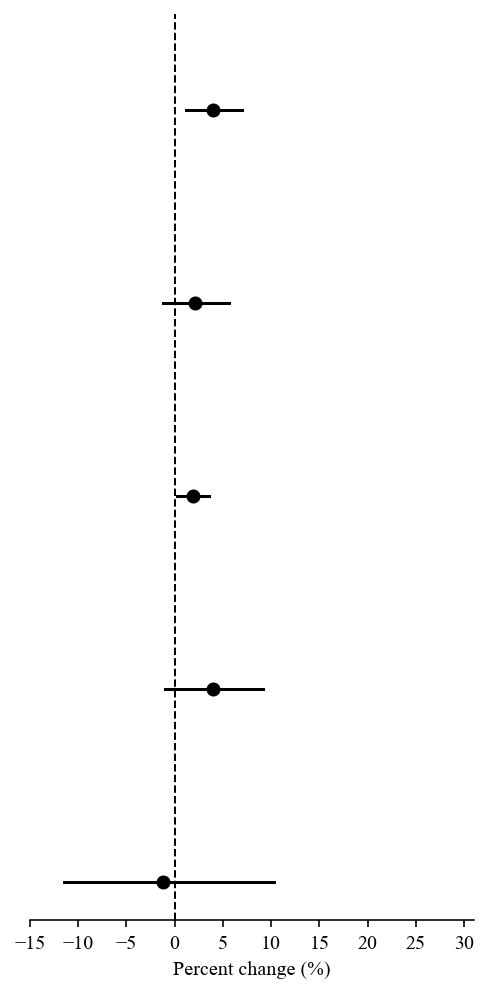 |
| Treated States | 42 | 1504 | 1499 | 4.02%  (1.16%, 6.96%) | 0.006 |  |
| Untreated States | 9 | 3629 | 3565 |  |  |  |
| **SUD inpatient** | | | | | |  |
| Treated States | 42 | 211 | 192 | 2.13%  (-1.26%, 5.63%) | 0.221 |  |
| Untreated States | 9 | 581 | 541 |  |  |  |
| **SUD medication** | | | | | |  |
| Treated States | 42 | 3386 | 3506 | 1.89%  (0.19%, 3.60%) | 0.029 |  |
| Untreated States | 9 | 7502 | 7432 |  |  |  |
| **OUD outpatient** | | | | | |  |
| Treated States | 42 | 623 | 631 | 3.96%  (-0.97%, 9.13%) | 0.117 |  |
| Untreated States | 9 | 1305 | 1306 |  |  |  |
| **OUD inpatient** | | | | | |  |
| Treated States | 42 | 31 | 28 | -1.17%  (-11.49%, 10.32%) | 0.834 |  |
| Untreated States | 9 | 93 | 87 |  |  |  |

***Notes:*** In the Phase 2 analysis, a standard difference-in-difference method is used for analysis. The negative binomial model is used for estimation. The treatment is the expiration of the federal eviction moratorium policy on August 26, 2021. The data sample includes 42 states in the treatment group and 9 states in the control group, with IL and WA censored periods after state-level eviction moratorium expiration. The study period is from week 23 to week 51 in 2021. States in the control group include those that continued to have state-level moratoriums after the expiration of the federal moratorium. The number of observations is 1,459 for each. State and year-month fixed effects are included.

**Appendix Table 16. Percent Change in Outcomes Attributable to Eviction Moratorium Expiration during Phase 1 (Mar 11th–Sep 1st), Using Wooldridge’s Difference-in-Differences Estimator**

|  | **N** | **Pre-period (mean)** | **Post-period (mean)** | **Percent Change**  **(95% CI)** | **P-value** | **Percent Change**  **(95% CI) (Graphical)** |
| --- | --- | --- | --- | --- | --- | --- |
| **SUD outpatient** | | | | | | **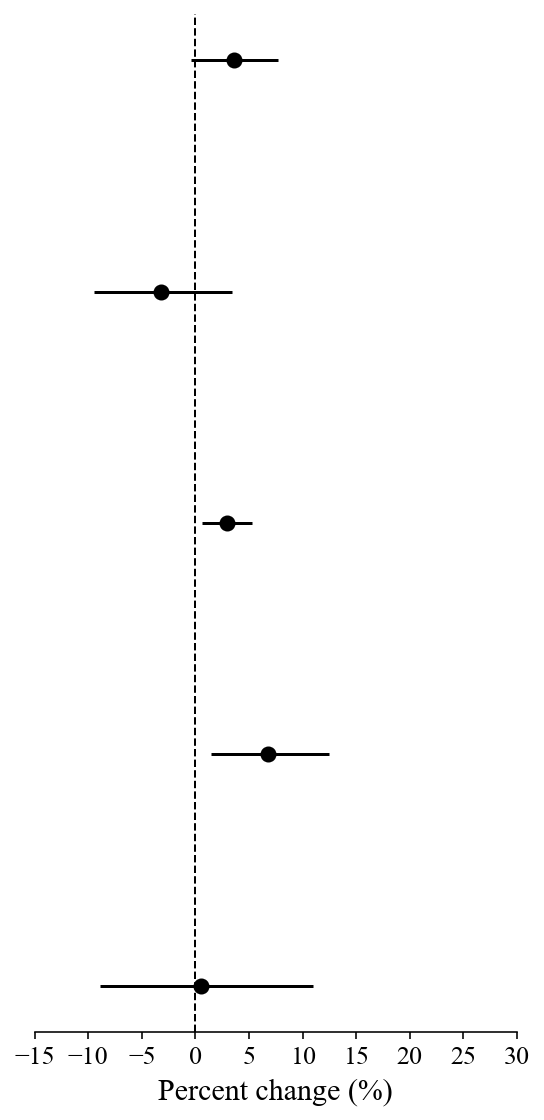** |
| Treated States | 26 | 1028 | 998 | 3.57%  (-0.39%, 7.68%) | 0.076 |  |
| Untreated States | 18 | 2419 | |  |  |  |
| **SUD inpatient** | | | | | |  |
| Treated States | 26 | 170 | 167 | -3.26%  (-9.48, 3.39%) | 0.320 |  |
| Untreated States | 18 | 390 | |  |  |  |
| **SUD medication** | | | | | |  |
| Treated States | 26 | 2609 | 2286 | 2.94%  (0.65%, 5.28%) | 0.013 |  |
| Untreated States | 18 | 5634 | |  |  |  |
| **OUD outpatient** | | | | | |  |
| Treated States | 26 | 450 | 411 | 6.79%  (1.41%, 12.47%) | 0.014 |  |
| Untreated States | 18 | 878 | |  |  |  |
| **OUD inpatient** | | | | | |  |
| Treated States | 26 | 24 | 22 | 0.55%  (-8.88%, 10.96%) | 0.911 |  |
| Untreated States | 18 | 59 | |  |  |  |

***Notes:*** In this Phase 1 analysis, Wooldridge’s difference-in-differences estimation method is used for analysis. The log version of the three outcomes is taken for estimation. The treatment is the expiration of the state-level eviction moratorium policy. The data sample includes 44 states having state-level eviction moratoriums. The study period is from week 11 to week 35 in 2020, starting from the week when the state’s eviction moratorium started. The treatment is the expiration of state-level eviction moratorium expiration of states in the treatment group. States in the control group include those that had state-level moratoriums but their moratorium did not expire during our observation period. The number of observations is 979 for each. State and year-month fixed effects are included.

**Appendix Table 17. Percent Change in Outcomes Attributable to Eviction Moratorium Expiration during Phase 1 (Mar 11th–Sep 1st), Using Gardner’s Two-Stage Difference-in-Differences Estimator**

|  | **N** | **Pre-period (mean)** | **Post-period (mean)** | **Percent Change**  **(95% CI)** | **P-value** | **Percent Change**  **(95% CI) (Graphical)** |
| --- | --- | --- | --- | --- | --- | --- |
| **SUD outpatient** | | | | | | **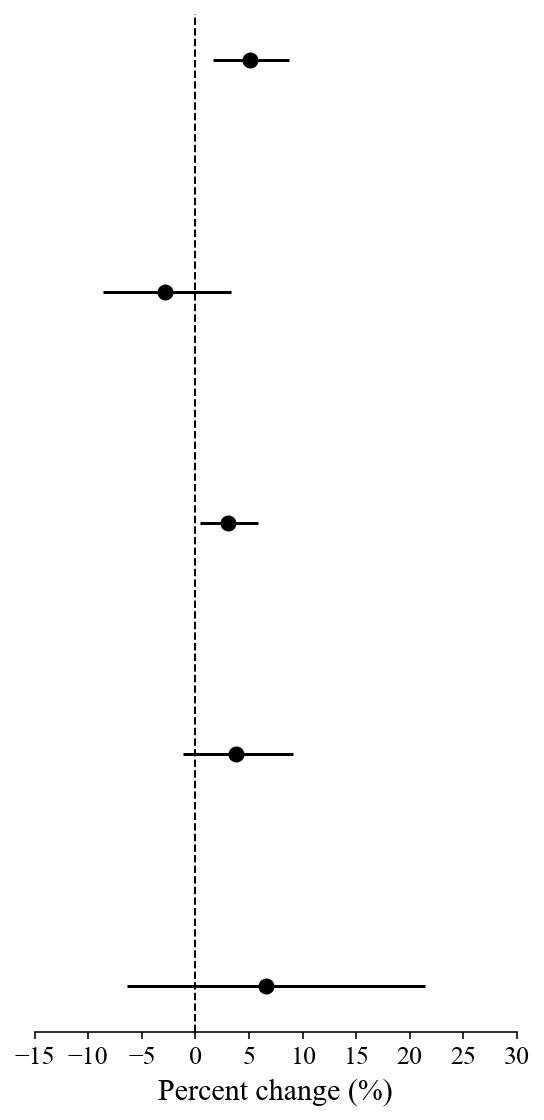** |
| Treated States | 26 | 1028 | 998 | 5.12%  (1.62%, 8.74%) | 0.004 |  |
| Untreated States | 18 | 2419 | |  |  |  |
| **SUD inpatient** | | | | | |  |
| Treated States | 26 | 170 | 167 | -2.85%  (-8.66%, 3.33%) | 0.358 |  |
| Untreated States | 18 | 390 | |  |  |  |
| **SUD medication** | | | | | |  |
| Treated States | 26 | 2609 | 2286 | 3.07%  (0.40%, 5.82%) | 0.024 |  |
| Untreated States | 18 | 5634 | |  |  |  |
| **OUD outpatient** | | | | | |  |
| Treated States | 26 | 450 | 411 | 3.83%  (-1.18%, 9.09%) | 0.136 |  |
| Untreated States | 18 | 878 | |  |  |  |
| **OUD inpatient** | | | | | |  |
| Treated States | 26 | 24 | 22 | 6.60%  (-6.37%, 21.39%) | 0.335 |  |
| Untreated States | 18 | 59 | |  |  |  |

***Notes:*** In this Phase 1 analysis, Gardner’s two-stage difference-in-differences estimation method is used for analysis. The log version of the three outcomes is taken for estimation. The treatment is the expiration of the state-level eviction moratorium policy. The data sample includes 44 states having state-level eviction moratoriums. The study period is from week 11 to week 35 in 2020, starting from the week when the state’s eviction moratorium started. The treatment is the expiration of state-level eviction moratorium expiration of states in the treatment group. States in the control group include those that had state-level moratoriums but their moratorium did not expire during our observation period. The number of observations is 979 for each. State and year-month fixed effects are included.

**Appendix Figure 1. Event Study Plot of SUD Outcomes in Phase One**


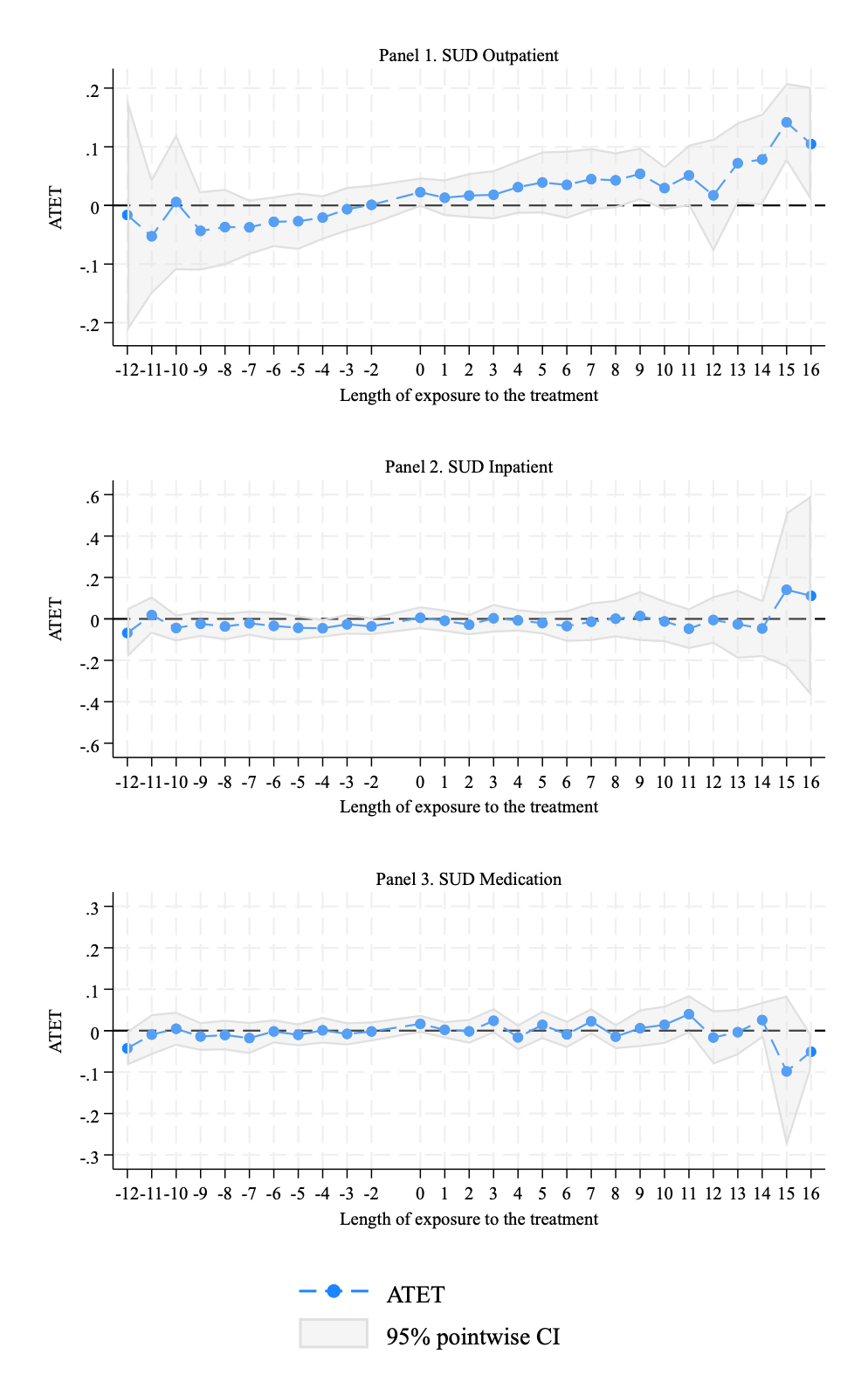


**Appendix Figure 2. Event Study Plot of OUD Outcomes in Phase One**


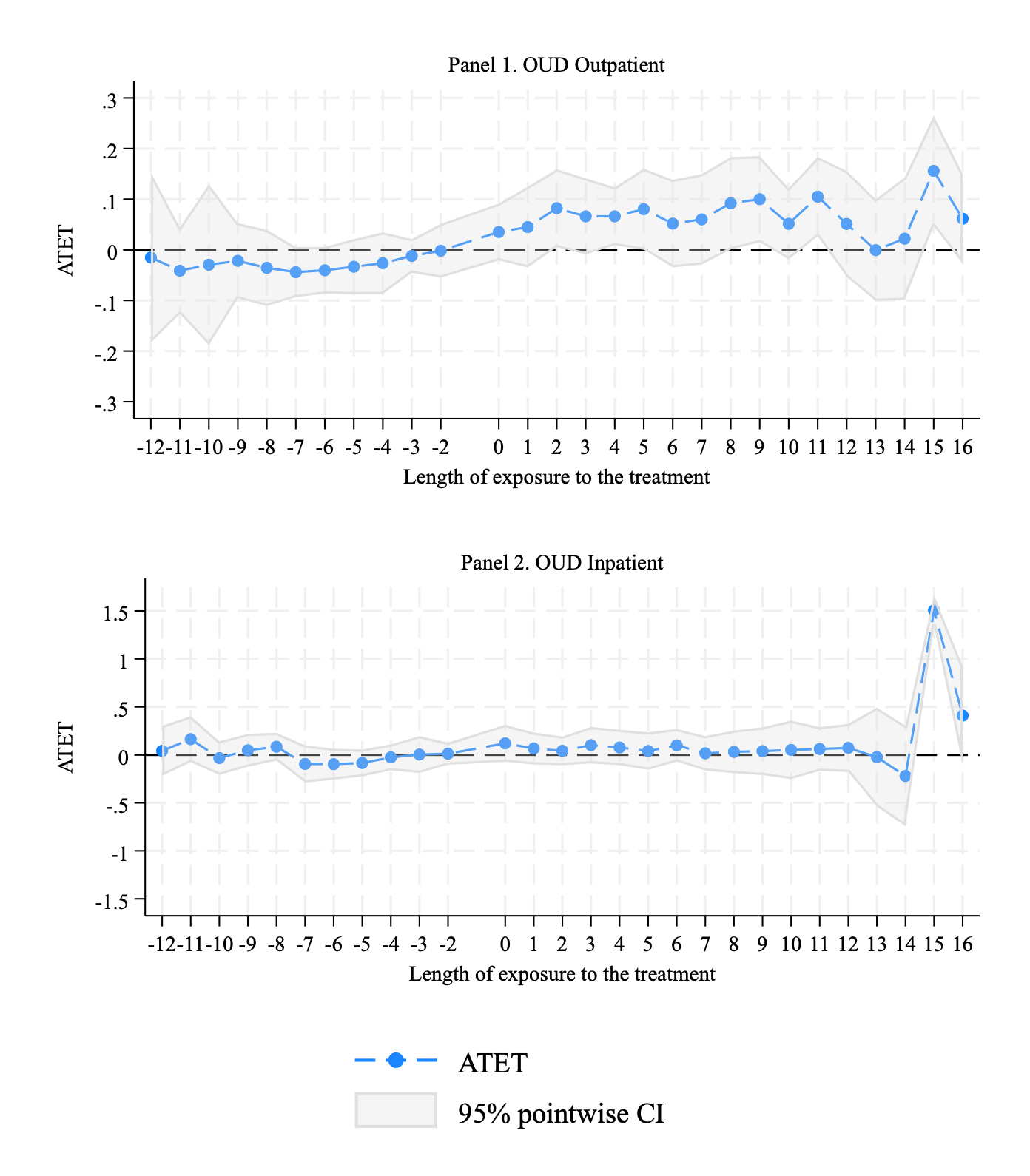


**Appendix Figure 3. Event Study Plot of SUD Outcomes in Phase Two**


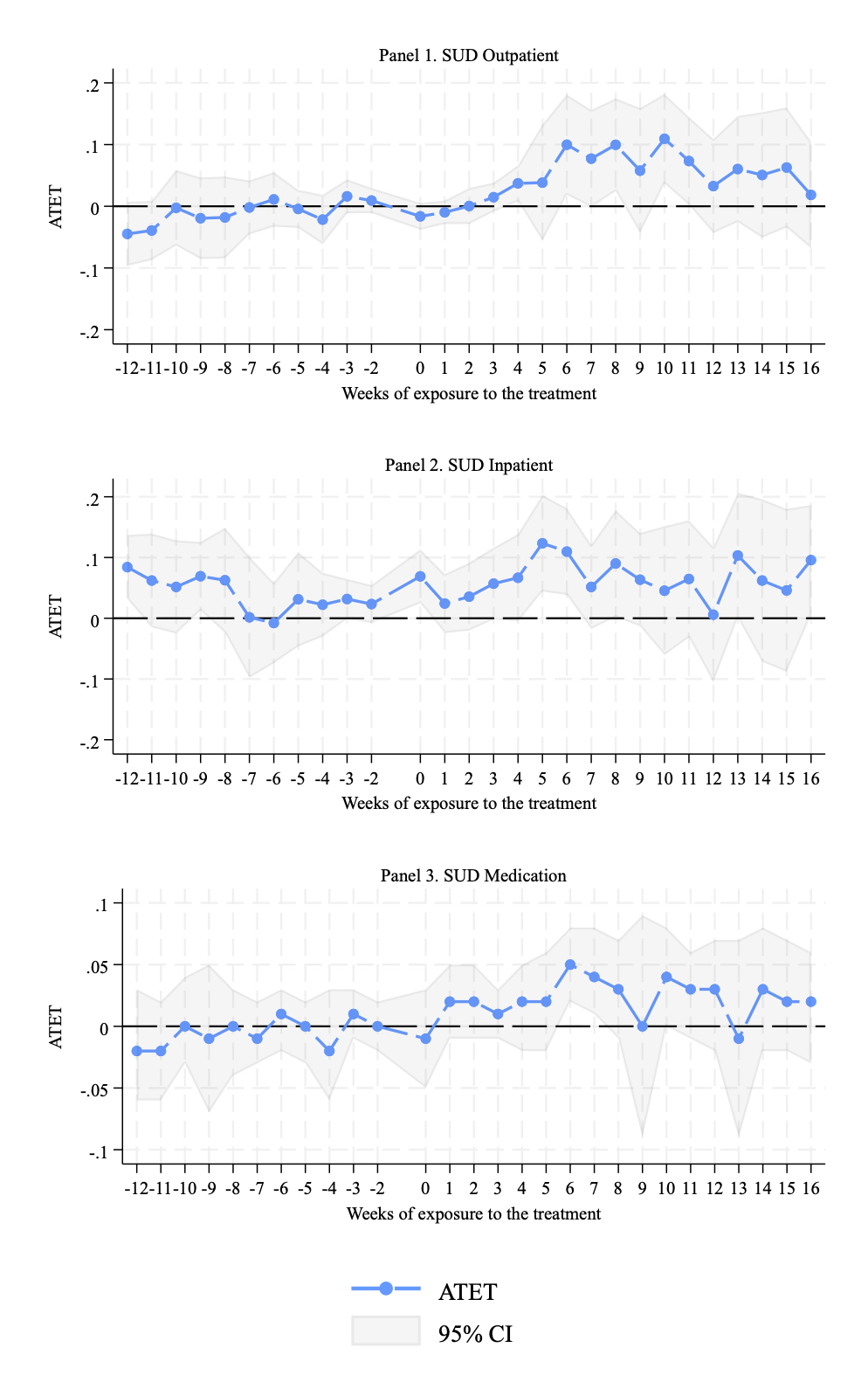


**Appendix Figure 4. Event Study Plot of OUD Outcomes in Phase Two**


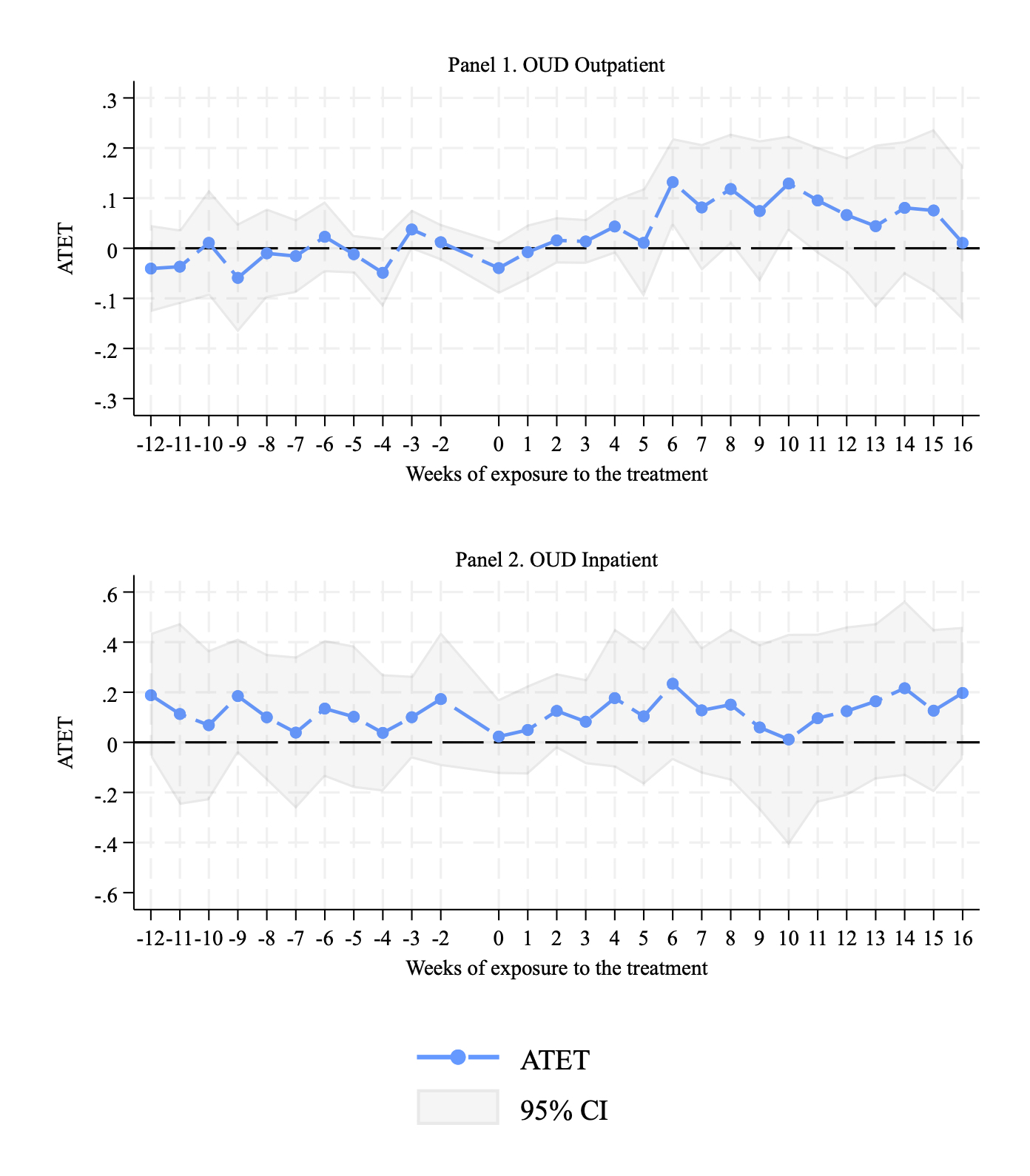

Supplement: qxag006_Supplementary_Data [file qxag006_supplementary_data.zip › Appendix.docx]
